# Histological and transcriptome analysis uncover a robust early PTI and ETI-associated immune response in *Musa acuminata* subsp. *burmannica* accession ‘Calcutta 4’ to *Fusarium oxysporum* f. sp. *cubense* Subtropical Race 4

**DOI:** 10.3389/fpls.2025.1621600

**Published:** 2025-09-03

**Authors:** Érica de Castro Costa, Lucas Santos Bastos, Andresa Priscila de Souza Ramos, Leandro de Souza Rocha, Edson Perito Amorim, Claudia Fortes Ferreira, Nicola Vergara Lopes Serão, Roberto Coiti Togawa, Priscila Grynberg, Robert Neil Gerard Miller

**Affiliations:** ^1^ Departamento de Biologia Celular, Universidade de Brasília, Brasília, DF, Brazil; ^2^ Embrapa Mandioca e Fruticultura, Cruz das Almas, BA, Brazil; ^3^ Departamento de Zootecnia, Universidade Federal de Lavras, Lavras, MG, Brazil; ^4^ Embrapa Recursos Genéticos e Biotecnologia, Brasília, DF, Brazil

**Keywords:** banana, Fusarium wilt, transcriptomics, genetic resistance, disease

## Abstract

**Introduction:**

Banana (*Musa spp*.) is a globally significant crop and a staple food in the diet of millions of people. However, commercial cultivars are highly susceptible to Fusarium wilt, a devastating disease caused by *Fusarium oxysporum* f. sp. *cubense (Foc)*. Tropical race 4 (TR4) and Subtropical race 4 (STR4) pose significant threats to banana production, including ‘Cavendish’ (AAA group), with STR4 pathogenic only in subtropical regions. Genetic resistance is the most effective strategy to combat Foc, underscoring the importance of advancing understanding of resistance mechanisms.

**Methods:**

Here, we identified and validated genes involved in the resistance response to Foc STR4 through RNA-seq and RT-qPCR analyses. Two genotypes were evaluated: ‘Calcutta 4’ (a resistant wild genotype, AA) and ‘Prata-Anã’ (a susceptible commercial genotype, AAB). Seedlings of ‘Calcutta 4’ and ‘Prata-Anã’ were inoculated with Foc STR4 isolate 218A, and root samples from ‘Calcutta 4’ were collected at 1, 2, and 4 days after inoculation (DAI) for RNA-seq analysis.

**Results:**

Comparative histological studies between the genotypes revealed defence responses, such as callose deposition and phenolic compound production, occurring exclusively in ‘Calcutta 4’ at 1 and 2 DAI, while colonization by *Foc* STR4 was observed only in ‘Prata-Anã’ at 8 and 15 DAI. RNA-seq analysis identified 1416 differentially expressed genes (DEGs) in ‘Calcutta 4’, based on comparisons between inoculated and non-inoculated control plants, log2FC >2 and <-2, and adjusted p-value for FDR at <0.05, with a rapid upregulation of 752 DEGs at 2 DAI, including genes associated with pattern recognition receptors, chitinases, phytohormones, resistance genes (from the NLR family), TFs, and systemic acquired resistance. Functional pathway analysis highlighted coordinated defence responses in ‘Calcutta 4’ to *Foc* STR4.

**Discussion:**

Together with functional validation of selected genes via RT-qPCR, these findings provide a foundation for the application of candidate genes in genetic improvement via introgression or gene-editing approaches. Given the close phylogenetic relationship between Foc STR4 and TR4, introgression of defense-related genes also holds promise for developing varieties that are resistant to both race 4 pathogens, relevant for mitigating the global impact of Fusarium wilt epidemics on banana production.

## 1 Introduction

Banana is globally the most widely cultivated, consumed, and exported fruit crop, with an annual production of 125 million tons (FAO, 2024). Together with plantains, they represent a staple food for over 400 million people globally (Voora et al., 2023), providing a source of carbohydrates, essential nutrients, vitamins A, B1, B2, B3, B6, and C, and minerals such as iron, potassium, phosphorus and calcium (Chandler, 1995). Banana cultivation faces numerous challenges that prevent further expansion of the international market (FAO, 2024). Diseases caused by phytopathogens are the primary cause of yield and quality losses, affecting plants at planting through to post-harvest and storage (Ghag et al., 2015). Among the fungal diseases, Yellow Sigatoka, caused by *Pseudocercospora musae* (Zimm.) Deighton, Black Sigatoka, caused by *P. fijiensis* (Morelet) Deighton, and Fusarium wilt, caused by *Fusarium oxysporum* f. sp. *cubense (Foc)* (E. F. Smith) Snyder & H.N. Hansen, are particularly destructive and can decimate entire banana plantations (Cordeiro et al., 2016; Ploetz et al., 2015). Fusarium wilt is a classic vascular wilt disease, where the pathogen enters the plant via direct penetration of the root epidermal cells, eventually reaching the vascular tissues. The first visible symptoms of disease include a discoloration of the rhizome and subsequent necrosis, with fungal colonization of vascular bundles in the pseudostem leading to xylem blockage. At this stage, the plant typically exhibits yellowing of the younger leaves and wilt symptoms. The period for appearance of symptoms can vary between two to six months, complicating early diagnosis of the disease (Cordeiro et al., 2016; Dita et al., 2018; Rishbeth, 1957). Disease progression will eventually cause death of the plant.

*Foc* is subdivided into three pathogenic races based on pathogenicity to different *Musa* cultivars. Race 1 affects ‘Gros Michel’ (AAA group), ‘Silk’, and ‘Pome’ (AAB group) cultivars; race 2 affects ‘Bluggoe’ (ABB group) (Ploetz, 2006); and race 4 is pathogenic to all cultivars, including ‘Cavendish’ (AAA group). Race 4 is further subdivided into Tropical Race 4 (TR4) and Subtropical Race 4 (STR4), with STR4 being less aggressive and capable of causing disease in ‘Cavendish’ only in subtropical regions (Buddenhagen, 2009). Conversely, TR4 comprises highly virulent isolates that cause disease in ‘Cavendish’ in both tropical and subtropical regions. An epidemic caused by *Foc* race 1 towards the end of the 19^th^ Century (Vézina, 2022a) devastated plantations of the susceptible ‘Gros Michel’ cultivar (AAA group), which was extensively cultivated in Central America at the time (Zheng et al., 2018). In Brazil, the first record of Fusarium Wilt occurred in 1930 in Piracicaba, São Paulo state, in the ‘Silk’ cultivar (AAB group), rapidly spreading throughout the country and decimating entire plantations (Cordeiro et al., 2016). The advance of the pathogen in banana production, especially in ‘Gros Michel’, prompted extensive research into the genetic improvement of *Musa* genotypes for resistance, resulting in the development of *Foc* race 1 - resistant ‘Cavendish’ cultivars (Buddenhagen, 1990). However, symptoms of Fusarium wilt were observed in ‘Cavendish’ cultivars in Taiwan in 1976, with the pathogen identified as *Foc* Tropical Race 4 (TR4) (Su et al., 1986). Rapid pathogen spread to ‘Cavendish’ plantations was subsequently reported in Asian countries including Indonesia and Malaysia (Pin, 1996) and China by the late 1990s (Vézina, 2022b). *Foc* TR4 was reported in Australia, Oman, Jordan, and Mozambique in 2015, in Pakistan and Lebanon in 2018, and in Vietnam, Laos, Myanmar, and Israel shortly thereafter (Nadiah Jamil et al., 2019; Maymon et al., 2020). The first case of *Foc* TR4 in Latin America was reported in Colombia in 2019 (García-Bastidas et al., 2019), followed by Peru in 2021 (Acuña et al., 2022), and Venezuela in 2023 (Mejías Herrera et al., 2023). In Brazil, *Foc* TR4 is a quarantine pathogen, regulated by the Ministry of Agriculture, Livestock and Food Supply. *Foc* STR4, meanwhile, infects ‘Cavendish‘ bananas in Australia, the Canary Islands, China, South Africa, and Taiwan (Munhoz et al., 2024), with widespread distribution in Brazil (Batista et al., 2022).

Banana production has declined over the past decade, primarily due to abiotic factors such as drought, heavy rainfall, and hurricanes, as well as biotic factors including pests and diseases. Despite the replacement of ‘Gros Michel’ with ‘Cavendish’ after *Foc* Race 1 devastation, one of the greatest challenges for the industry, exacerbated by climate change, is the increased spread of diseases (Voora et al., 2023). Salvacion et al. (2019), for example, estimated that climate change could expand areas affected by *Foc* by 67% in a study in the Philippines. Globally, Fusarium wilt caused by all pathogenic races currently results in an annual yield loss of 60–90% (Sankari et al., 2023). In Brazil, disease incidence in ‘Silk’ fields has reached up to 41.42%, with a production impact of 1856.7 kg ha^-^¹ year^-^¹, translating to an average loss of USD 109.8 ha^-^¹ year^-^¹ (Heck et al., 2021).

Considering that *Foc* chlamydospores can persist in the soil for over 30 years in the absence of a host (Stover, 1962; Ploetz, 2006; Pegg et al., 2019), the most effective management strategy to control Fusarium wilt is via exclusion, preventing the introduction of the pathogen into banana-producing areas. Chemical, cultural, and biological control methods for managing Fusarium wilt, by contrast, have not yet proven to be sufficiently effective to protect susceptible cultivars (Ploetz et al., 2015). Given this scenario, the only effective control method, when the pathogen is present in production fields, is to employ resistant cultivars against *Foc* (Xu et al., 2011; Ploetz, 2015; Kema et al., 2021). Whilst vegetative propagation of sterile commercial bananas equates to a low genetic variability, increasing vulnerability to rapidly evolving pathogen races (Simmonds, 1953; Silva et al., 2013, 2016), greater genetic variability can be found in wild subspecies of *M. acuminata* and their varieties. The *M. acuminata* subsp. *burmannica* accession ‘Calcutta 4’, which originates from Southeast Asia at the *Musa* center of origin, has co-evolved with several pathogens and, as a result, has developed an arsenal of defense mechanisms to biotic stress. This accession is widely used in breeding programs to develop disease-resistant cultivars (Miller et al., 2010; Sachter-Smith, 2023).

To combat recurring outbreaks, research is also increasingly focused on understanding the genetic mechanisms underlying host resistance to *Foc*, with focus on candidate gene discovery including resistant wild bananas such as *M. acuminata* ‘Pahang’ and *M. acuminata* subsp. *burmannica* ‘Calcutta 4’ (Miller et al., 2008; Kotari et al., 2018; Zhang et al., 2018, 2019; Chen et al., 2019). Genes expressed in wild *Musa* genotypes during interactions with *Foc* have been applied to enhance resistance in commercial cultivars. Genes such as *MusaDAD1*, *MusaBAG1*, and *MusaBI1*, which regulate cell death, are efficient after cisgenesis in banana cv. ‘Rasthali’ (AAB subgroup), with minimum symptom development after inoculation with *Foc* race 1 (Ghag et al., 2014). Through introgression of an NLR *RGA2* gene from a wild species, together with the *Ced9* gene from *Caenorhabditis elegans*, which plays a crucial role in the regulation of apoptosis, Dale et al. (2017) also transformed Cavendish cv. ‘Grande Naine’, with cisgenic plants exhibiting high resistance to *Foc* TR4, including in field trials. In a subsequent advancement, QCAV-4, containing a single *RGA2* transgene, was approved in 2025 by Australian regulatory authorities for commercial cultivation and human consumption, representing the first genetically modified ‘Cavendish’ authorized for such use (Harding et al., 2025).

Cultivars such as ‘Pome’ (AAB), ‘Silk’ (AAB) and ‘Gros Michel’, all of which are susceptible to *Foc* races 1 STR4 and TR4, predominate in local Latin American and Caribbean markets (Munhoz et al., 2024). Given the widespread presence of STR4 in the region, the aim of this study was to identify genes involved in the defense response of *M. acuminata* subsp. *burmannica* accession ‘Calcutta 4’ (AA), during interaction with the pathogen *F. oxysporum* f. sp. *cubense* race STR4. RNA-seq-based analysis of differential gene expression together with investigation of structural defense responses provided insights into the immune response in this resistant wild diploid. Data serve as a basis for future introgression of defense genes into commercial varieties such as ‘Prata’, ‘Silk’ and ‘Cavendish’.

## 2 Materials and methods

### 2.1 Preparation of *Musa* seedlings

*In vitro*-propagated seedlings of the wild accession ‘Calcutta 4’ (*Musa acuminata* subsp. *burmannica*, subgroup AA), resistant to *F. oxysporum* f. sp. *cubense* race 1 and 4 (STR4 and TR4), and the susceptible cultivar to both races ‘Prata-anã’ (*Musa* sp., subgroup ‘Pome’, AAB), were obtained from the Embrapa Cassava and Fruits Germplasm Bank (Cruz das Almas, BA, Brazil). Seedlings were transplanted to sterile coconut fiber substrate and acclimatized in a greenhouse at 24°C with irrigation every 48 h. After 30 days, 100 mL per plant of a 1g/L monoammonium phosphate (MAP) solution was applied. After 45 days, plants were transferred to 2L pots with fresh sterile coconut fiber. A second fertilization was applied after 30 days using a nutrient mixture of urea, magnesium sulfate, MAP, calcium sulfate, and potassium sulfate (1 g/L each), followed 10 days later with a final fertilization with zinc sulfate (1 g/L).

### 2.2 Inoculum preparation

*F. oxysporum* f. sp. *cubense* STR4 strain 218A, isolated from Nanica (‘Cavendish’) plants in São Paulo, Brazil and classified as VCG 0120, recognized as *Foc* STR4 (Rocha et al., 2018), was maintained at Embrapa Cassava and Fruits. Inoculum was prepared using rice infested with 10^6^ CFU/g of *Foc* STR4 218A, according to Santana et al. (2024).

### 2.3 Bioassays

‘Calcutta 4’ and ‘Prata-anã’ plants were inoculated by adding 50g of *Foc* STR4 218A-infested rice to the coconut fiber substrate; controls received non-infested rice. The experiment followed a completely randomized design, with each treatment comprising three independent biological replicates. Total roots from inoculated and non-inoculated control plants were collected at 1, 2, and 4 days after inoculation (DAI) for RNA-seq and RT-qPCR, flash-frozen in liquid nitrogen, and stored at -80°C.

### 2.4 Histological analyses

Root samples from both genotypes were collected at 1, 2, 4, 8, and 15 DAI. Root fragments collected at 1, 2, and 4 DAI were analyzed for callose and phenolic compound deposition following Rocha et al. (2022).

For scanning electron microscopy (SEM), root samples from the same timepoints were post-fixed in 1% osmium tetroxide (1h), dehydrated in an acetone gradient (30%, 50%, 70%, 90%, and 100%), dried in a critical point dryer (Blazers CPD 030), gold-coated using a sputter coater (Leica EM SCD 500), and visualized using a scanning electron microscope (JEOL JSM 7001F).To assess *Foc* STR4 colonization of host tissues, root fragments collected at 1, 2, 4, 8, and 15 DAI from ‘Calcutta 4’ and ‘Prata-anã’ were assessed through clearing and staining according to Rocha et al. (2022).

### 2.5 Symptom evaluation

Seedlings of both genotypes were inoculated with *Foc* STR4 218A (10^6^ CFU/g rice), with non-inoculated controls as per Santana et al. (2024). Wilt symptoms, leaf yellowing, and rhizome necrosis, were recorded as present or absent at 90 and 120 DAI.

### 2.6 Total RNA extraction

Roots from ‘Calcutta 4’ and ‘Prata-anã’ at 1, 2, and 4 DAI were flash-frozen in liquid nitrogen and stored at -80°C. Root fragments were ground in liquid nitrogen, and total RNA was extracted using 1 mL of PureLink™ Plant RNA Reagent (Invitrogen – ThermoFisher Scientific, Waltham, MA, USA) per sample for 5 min, followed by centrifugation (10 min, 15,000 rpm, 4°C). The supernatant was purified using the Direct-zol™ RNA Miniprep Plus Kit (Zymo Research, Irvine, CA, USA) following the manufacturer’s instructions. RNA integrity was verified by agarose gel electrophoresis and quantified via NanoDrop™ One Microvolume spectrophotometry (ThermoFisher Technologies, Waltham, MA, USA).

### 2.7 RNA-seq library preparation and sequencing

Total RNA from ‘Calcutta 4’ at 1, 2, and 4 DAI, and from non-inoculated controls, was stabilized using RNA stable™ (Biomatrica, San Diego, CA, USA) and sequenced at the Genome Quebec Innovation Center, Canada. RNA-Seq libraries were constructed using the mRNA stranded Library Prep Kit (Illumina Inc., San Diego, CA, USA) and sequenced as paired-end reads (2x100 bases) on an Illumina NovaSeq 6000 system (Illumina Inc., San Diego, CA, USA).

### 2.8 RNA-seq data processing and differential expression analysis

Raw paired-end reads were quality-checked with FastQC (FastQC - https://www.bioinformatics.babraham.ac.uk/projects/fastqc/)), then filtered (Fastq QC>30 and minimum length ≥ 100 bp following trimming and adapter removal) using Trimmomatic (Bolger et al., 2014). High-quality reads were aligned in batch mode to the *M. acuminata* DH-Pahang v.4 reference genome (available at the Banana Genome HUB: https://banana-genome-hub.southgreen.fr/organism/Musa/acuminata), using the STAR software in two-pass Basic Mode (Dobin et al., 2013). Multi-mapped reads were excluded from downstream analyses. Quantification of reads mapping to annotated gene models was conducted using HTSeq-count (Python Software Foundation, Portland, OR, USA) (Anders et al., 2015).

Differential expression analysis was performed using EdgeR’s exact test in Rstudio (Robinson et al., 2010). Differentially expressed genes (DEGs) were identified based on significance determined based on adjusted p-values (FDR ≤ 0.05) using the Benjamini–Hochberg procedure to control the false discovery rate, with the log2 Fold Change (log2FC) employed for determining expression changes between treatments [‘Calcutta 4’-inoculated (I) vs. non-inoculated (NI)].

### 2.9 Functional analysis of DEGs

Multiple functional annotation strategies were employed to identify key processes and metabolic pathways affected by biotic stress, including Gene Ontology (GO), the Kyoto Encyclopedia of Genes and Genomes (KEGG - https://www.genome.jp) (Kanehisa et al., 2016a), EuKaryotic Orthologous Groups (KOG) (Tatusov et al., 2003), and MapMan (Thimm et al., 2004). GO annotation of DEGs was performed by homology using GO enrichment software (https://banana-genome-hub.southgreen.fr/content/go-enrichment), with parameters “Merge similar GO-term: sensitive”, p-value ≤0.05, and q-value ≤0.01 (Droc et al., 2013). For KEGG pathway mapping, DEG protein sequences were analyzed with BlastKOALA (https://www.kegg.jp/blastkoala/) to generate KEGG identifiers (KOs) based on sequence similarity to the KEGG gene database subset (Kanehisa et al., 2016b). Generated KOs were then used to construct KEGG pathway maps (https://www.genome.jp/kegg/mapper/) (Kanehisa and Sato, 2020; Kanehisa et al., 2022). KOG annotation was performed using eggNOG v5.0 (http://eggnog5.embl.de) (Huerta-Cepas et al., 2019) based on sequence similarity. For MapMan pathway analysis (v. 3.5.1) (Thimm et al., 2004), DEG protein sequences were hierarchically annotated using Mercator4 v.5 (Lohse et al., 2014) and mapped to the *M. acuminata* gene matrix, along with corresponding Log2FC expression values.

Additionally, InterPro (https://www.ebi.ac.uk/interpro/) (Mitchell et al., 2019) and InterProScan (Jones et al., 2014) were employed to assign functional domains to DEG protein products, providing insight into the protein families and domains involved.

### 2.10 Validation of candidate genes via RT-qPCR

Fifteen DEGs were selected for expression validation via reverse transcription quantitative real-time PCR (RT-qPCR) (**Table 1**). Primers targeting the transcript sequence of each gene were designed using the PrimerQuest Tool (IDT - https://www.idtdna.com/pages/tools/primerquest), with the following parameters: amplicon size 80–150 bp, primer length 18–25 nt, GC content 40-60%, and melting temperature 57 - 63°C (ideally 60°C). Primer specificity was verified using NCBI Primer-BLAST for eukaryotic organisms (https://www.ncbi.nlm.nih.gov/tools/primer-blast). For each treatment, for both ‘Calcutta 4’ and ‘Prata-anã’, total RNA was Dnase-treated as described previously (Section: Total RNA extraction). cDNA synthesis was conducted using the SuperScript™ IV First-strand Synthesis System with Oligo(DT) primers (Invitrogen Thermo Fisher Scientific, Waltham, MA, USA) following the manufacturer’s protocol, using RNA from each sample at a standardized initial concentration of 100 ng/μL. For RT-qPCR validation of target genes, cDNA was diluted 1:20 from stock solutions. Each 10 µL reaction comprised 0.2 µM of each primer (forward and reverse), 5 µL of iTaq Universal SYBR Green Supermix (BioRad, Hercules, CA, USA), 2 µL of diluted cDNA, and 2.6 µL of ddH_2_O. Stable reference genes L2 and bTUB3 were employed for ‘Calcutta 4’, and ACTIN1 and GAPDH for ‘Prata-anã’, as previously described for these two pathosystems by Costa et al. (2024). Thermocycling was performed on an ABI StepOne Plus Real-Time Thermocycler (Applied Biosystems, Thermo Fisher Scientific, Waltham, MA, USA) with three independent biological and three technical replicates per reaction. Cycling conditions were as follows: initial denaturation at 50°C for 2 min, 95°C for 10 min; 40 cycles of 95°C for 15 s and 60°C for 60 s. Melting curves were generated in three steps: 95°C for 15 s, 60°C for 60 s, and 95°C for 15 s.

**Table 1:** Differentially expressed genes (DEGs) identified from the *in silico* RNA-seq analysis of ‘Calcutta 4’ following infection with *Foc* STR4 at 1, 2, and 4 DAI, selected for validation via RT-qPCR.

Gene	Product	Primer F	Primer R
Macma4_01_g21300	Acidic endochitinase	GGACTAGATACCACGACAGA	AGACTTCACCGGCATAGA
Macma4_02_g09450	Leucine-rich repeat receptor-like serine/threonine/tyrosine-protein kinase SOBIR1	TACGGATCGCCATCTTCT	GGCTTTGGCTGTTAGTGT
Macma4_03_g08040	Lipoxygenase 3-2C chloroplastic	CCTCCTGTGAGTAAGCTAGA	GCAGAGACATGCCATTAAGA
Macma4_03_g29630	Basic endochitinase C	GGACTGCTACAACCAGAAAC	CGGAGTCGCTATCTCAGTAA
Macma4_04_g03250	Salicylic acid-binding protein 2	TGTCATCGAGACCTCCTTT	ACACATCTGCGGCATTAC
Macma4_04_g37480	putative Disease resistance protein RPS5	CACTATGGGATCTCGGAATTG	GACGGCTTGGGAGTATATTG
Macma4_06_g16370	Peroxidase 4	GACACTACTGTCCCTTGATTAC	GTGGCAGTATCATCCAGAAG
Macma4_06_g30880	putative L-type lectin-domain containing receptor kinase IX.1	CGGGTTTGCCTTCTTCTT	CGTGGTGTCGTTGGATAAA
Macma4_07_g21750	Premnaspirodiene oxygenase	GAACAAAGGACGGCAAGTA	CAGGAAGTAGCACTCACAAG
Macma4_09_g31980	NDR1/HIN1-like protein 10	GTGTGATGTGGATCTCTTCTG	GAGACAGTCTGCTGCTAAAC
Macma4_10_g08320	Nematode resistance protein-like HSPRO2	AATCGAGCGGAGGATACA	TCTATCAGCATGTGGAGGT
Macma4_10_g17470	Cysteine-rich repeat secretory protein 55	GGTGCGCTACGAGATTTAT	AACAAGATGGCCTATGGATG
Macma4_10_g24620	Stilbene synthase 1	GATCGAGTCAGCAAGCATAG	AAGCGAGGAAACAGAAAGG
Macma4_08_g16850	Protein SAR DEFICIENT 1	CTAACGGAGAGGTTCCTTTATC	GGTCCACTCATTTCCATCTC
Macma4_03_g08420	Pathogenesis-related protein 1	GGGTACGTGAAGGAAAGATTGG	TCGGCTCGAACTTGAAATGG
Macma4_05_g17130	Thaumatin-like protein 1	GATGCGACGCTGATGAAA	AGACCGGCCATAAGATACA

### 2.11 Statistical analysis of RT-qPCR data

The qPCR data were analyzed according to the linear mixed model below, following the strategy proposed by Steibel et al. (2009). Data on the target and endogenous control genes are analyzed simultaneously:


$$\begin{array}{c} CT_{ijklm}=\mu +G_{i}+P{\left(G\right)}_{\left(i\right)j}+T_{k}+{\left(GT\right)}_{ik}+[P\left(G\right)T{]}_{\left(i\right)jk}+D_{l}\\ +{\left(GD\right)}_{ik}+[P\left(G\right)D{]}_{\left(i\right)jl}+{\left(TD\right)}_{kl}+{\left(GTD\right)}_{ikl}+{\left[P\left(G\right)TD\right]}_{\left(i\right)jkl}\\ +S_{m}+e_{ijklm} \end{array}$$

where $CT_{ijklm}$ is the CT value; μ is the overall mean, $G_{i}$ is the fixed effect of the *i* ^th^ level of Genotype, with *i* = 1 or 2; $P{\left(G\right)}_{\left(i\right)j}$ is the fixed effect of the *j* ^th^ level of Primer (target/endogenous) within Genotype, with *j* = 1 to 3; $T_{k}$ is the fixed effect of the *k* ^th^ level of Treatment, with *k* = 1 or 2; $D_{l}$ is the fixed effect of the *l* ^th^ level of Day, with *k* = 1 to 3; ${\left(GT\right)}_{ik}$ , ${\left[P\left(G\right)T\right]}_{\left(i\right)jk}$ , ${\left(GD\right)}_{ik}$ , ${\left[P\left(G\right)D\right]}_{\left(i\right)jl}$ , ${\left(TD\right)}_{kl}$ , ${\left(GTD\right)}_{kl}$ , and ${\left[P\left(G\right)TD\right]}_{\left(i\right)jkl}$ , represent the fixed effect interactions among the previously described parameters; $S_{m}$ is the random effect of the *m* ^th^ Sample, assuming $S\sim N\left(0,I_{s}\sigma_{s}^{2}\right)$ , where $I_{s}$ represents the identity matrix with dimensions equal to the number of samples and $\sigma_{s}^{2}$ the sample variance; and $e_{ijklm}$ is the random error associated with $CT_{ijklm}$ , assuming $e\sim N\left(0,I_{e}\sigma_{e}^{2}\right)$ , where $I_{e}$ represents the identity matrix with dimensions equal to the total number of observations.

Orthogonal contrasts were constructed for model terms including the effect of Primer to obtain *P*-values for effect of interest: $P{\left(G\right)}_{\left(i\right)j}$ for the main effect of Genotype, ${\left[P\left(G\right)T\right]}_{\left(i\right)jk}$ for the main effect of Treatment and Genotype-Treatment interaction, ${\left[P\left(G\right)D\right]}_{\left(i\right)jl}$ for the main effect of Day and Genotype-Day interaction, and ${\left[P\left(G\right)TD\right]}_{\left(i\right)jkl}\;$ for the Genotype-Treatment-Day interaction. Similarly, contrasts were used to compute −ΔadjCT as the difference in CTs between the target and the average of the two endogenous genes, and −ΔΔCT for pairwise comparisons of interest. This analysis enables the appropriate modelling of all sources of variation, based on the assumption of a Gaussian distribution for log-transformed expression levels, utilizing heteroscedastic models that allow for heterogeneous variances and providing a more realistic and precise fit for gene expression data. Additionally, the inclusion of sample-specific effects, representing the total mRNA level, enhances the accuracy and applicability of the results obtained. All analyses were performed in SAS Studio 3.81 (Enterprise Edition, SAS Institute Inc., Cary, NC, USA). The −ΔΔCT and Log2FC values were used to calculate relative expression values using the $2^{-\Delta \Delta CT}$ method (Livak and Schmittgen, 2001). These values were then employed in graphical representations generated using the R software (R Core Team, 2023), specifically the ggplot2 package (Wickham, 2016).

## 3 Results

### 3.1 Histological analysis of the host-pathogen interactions in ‘Calcutta 4’ and ‘Prata-anã’

**Figure 1:**
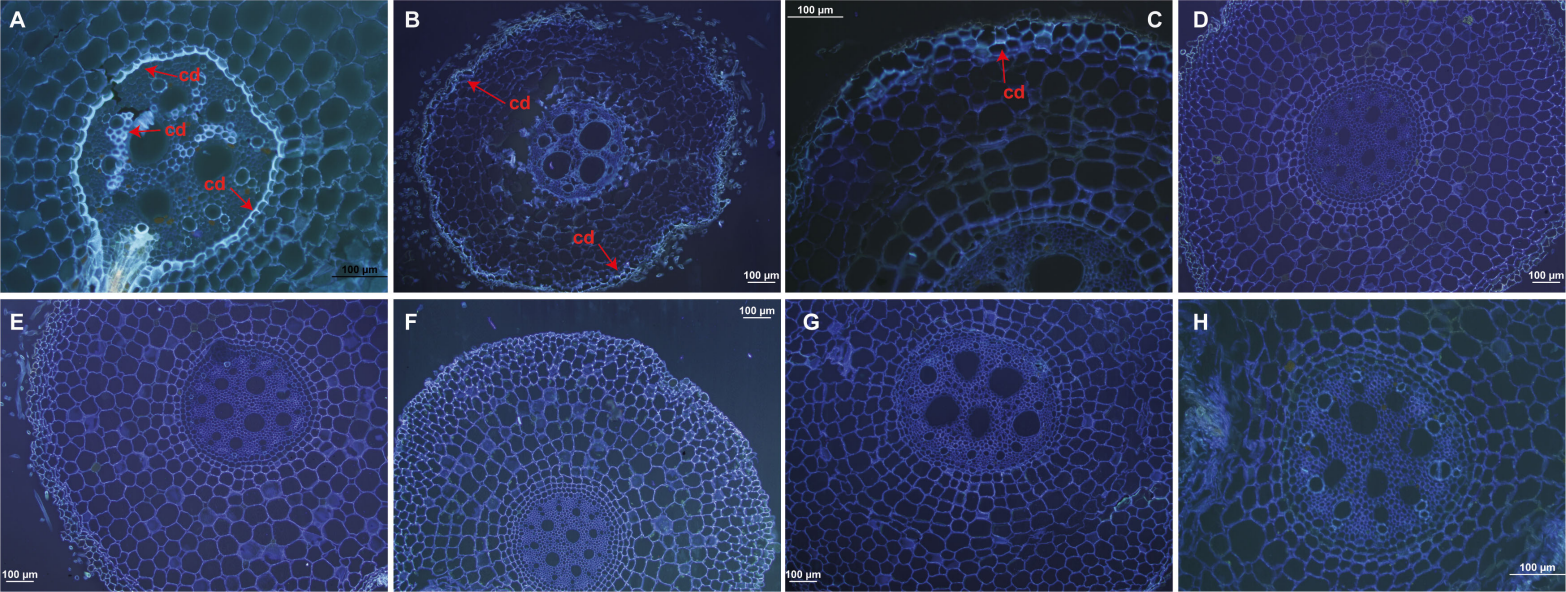
Histological analyses of callose deposition in root tissues in ‘Calcutta 4’ (C4) and ‘Prata-anã’ (PA). **(A-C)** Callose deposition analysis in C4 at 1, 2, and 4 DAI, respectively; **(D-F)** Callose deposition analysis in PA at 1, 2, and 4 DAI, respectively; **(G, H)** Non-inoculated controls for C4 and PA. Arrows highlight fluorescence, indicative of callose deposition (cd). (Bar = 100 µm).

**Figure 2:**
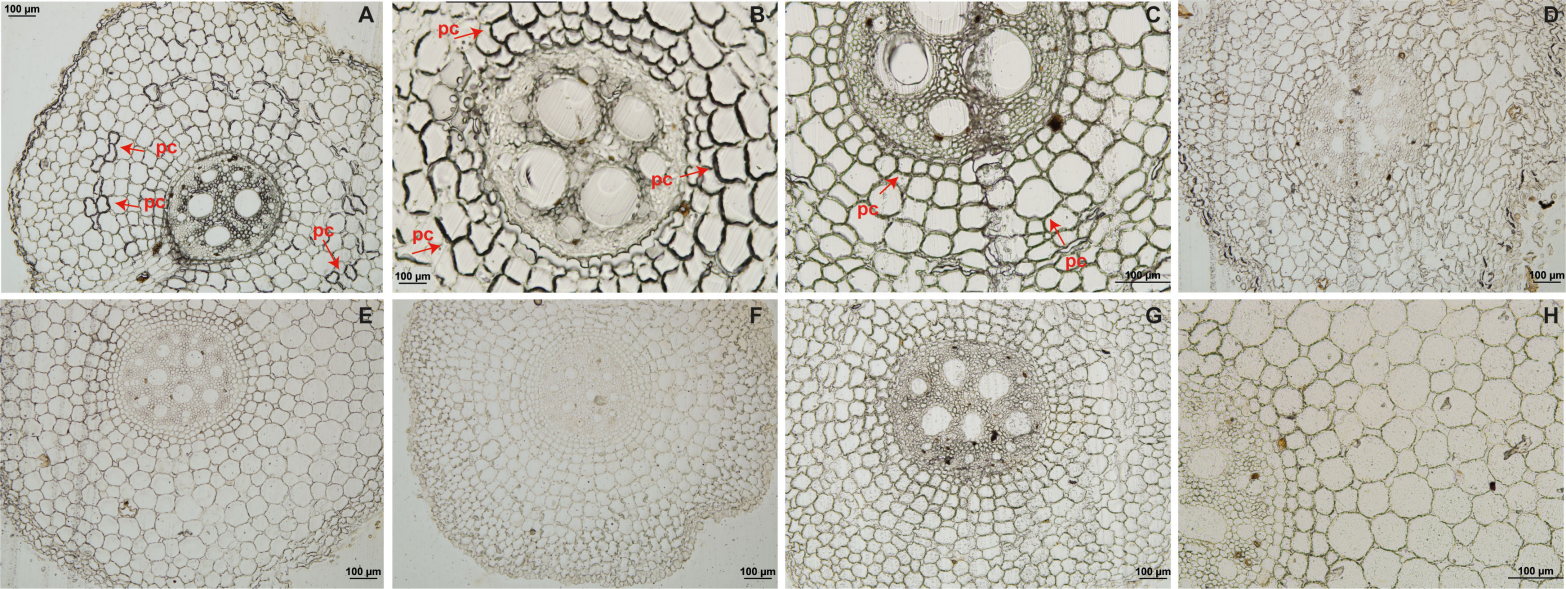
Histological analyses of phenolic compound deposition in root tissues in ‘Calcutta 4’ (C4) and ‘Prata-anã’ (PA). **(A-C)** Phenolic compound analysis in C4 at 1, 2, and 4 days after inoculation (DAI), respectively; **(D-F)** Phenolic compound analysis in PA at 1, 2, and 4 DAI, respectively; **(G, H)** Non-inoculated controls for C4 and PA. Arrows indicate the presence of phenolic compounds (pc). (Bar = 100 µm).

Root sections from resistant ‘Calcutta 4’ inoculated with *Foc* STR4 218A showed the presence of callose deposition at 1 DAI, indicated by a strong fluorescence in the root cortex cells (**Figure 1A**). Lower fluorescence was observed at 2 and 4 DAI (**Figures 1B, C**), with no fluorescence observed in the non-inoculated control treatment (**Figure 1G**). In susceptible ‘Prata-anã’, by contrast, neither inoculated or non-inoculated control samples showed any evidence for callose deposition in the stained root sections (**Figures 1D–F, H**). A similar pattern of physiological responses was observed regarding the formation of phenolic compounds. Parenchymal cells in ‘Calcutta 4’ root fragments revealed deposition of phenolic compounds at 1 and 2 DAI (**Figures 2A, B**), followed by a reduction at 4 DAI (**Figure 2C**). No phenolic compound deposition was observed in C4 non-inoculated controls (**Figure 2G**). In the case of the susceptible genotype ‘Prata-anã’, none of the investigated time points or controls revealed evidence for phenolic compound formation (**Figures 2D–H**).

**Figure 3:**
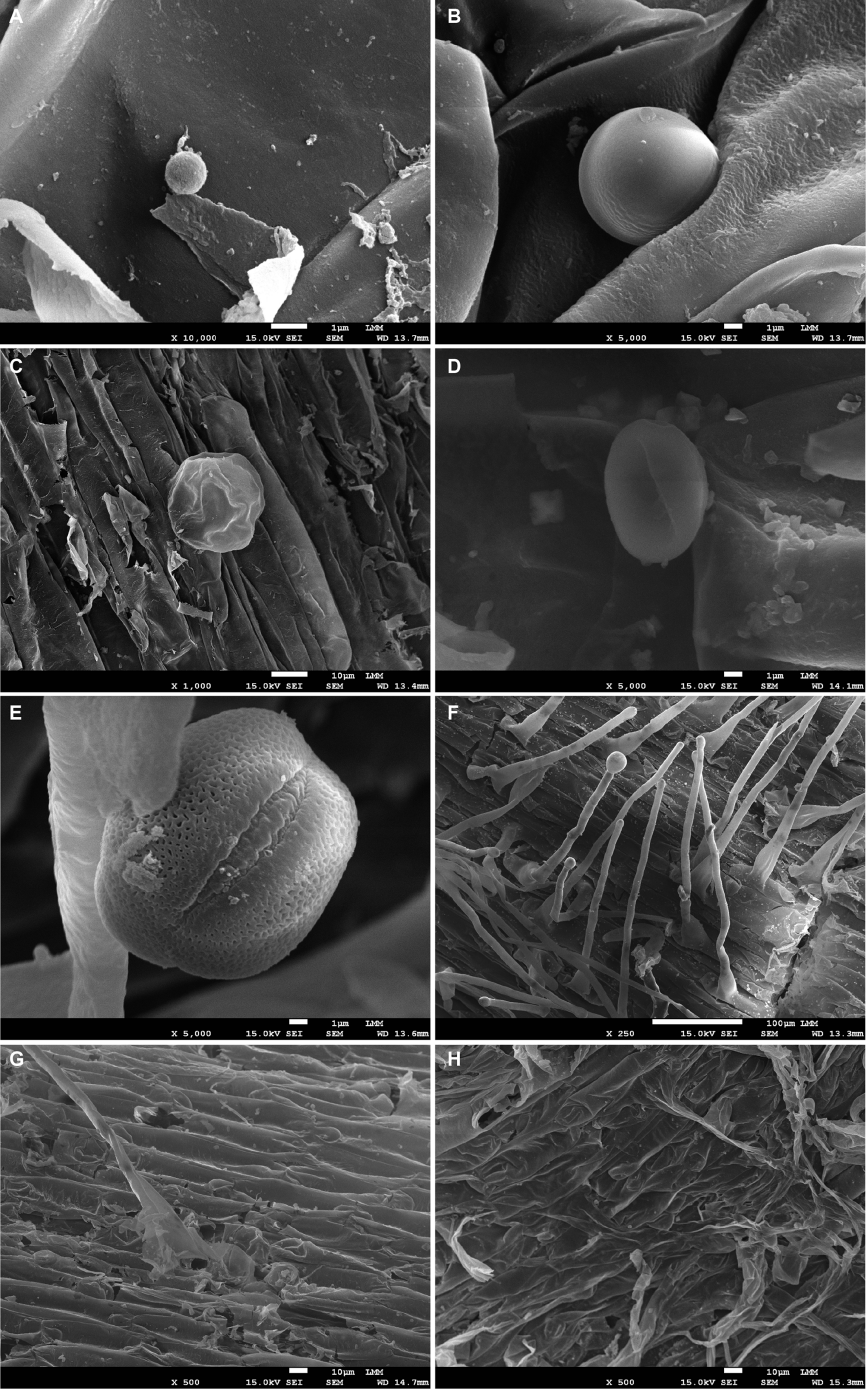
Scanning microscopy analysis of roots from ‘Calcutta 4’ (C4) **(A-C)** and ‘Prata-anã’ (PA) **(D-F)** inoculated at 1, 2, and 4 days after inoculation (DAI), respectively. **(G)** non-inoculated C4 control; **(H)** non-inoculated PA control. Scale bars: **(A, B, D, E)** = 1 µm; **(C, G, H)** = 10 µm; **(F)** = 100 µm.

**Figure 4:**
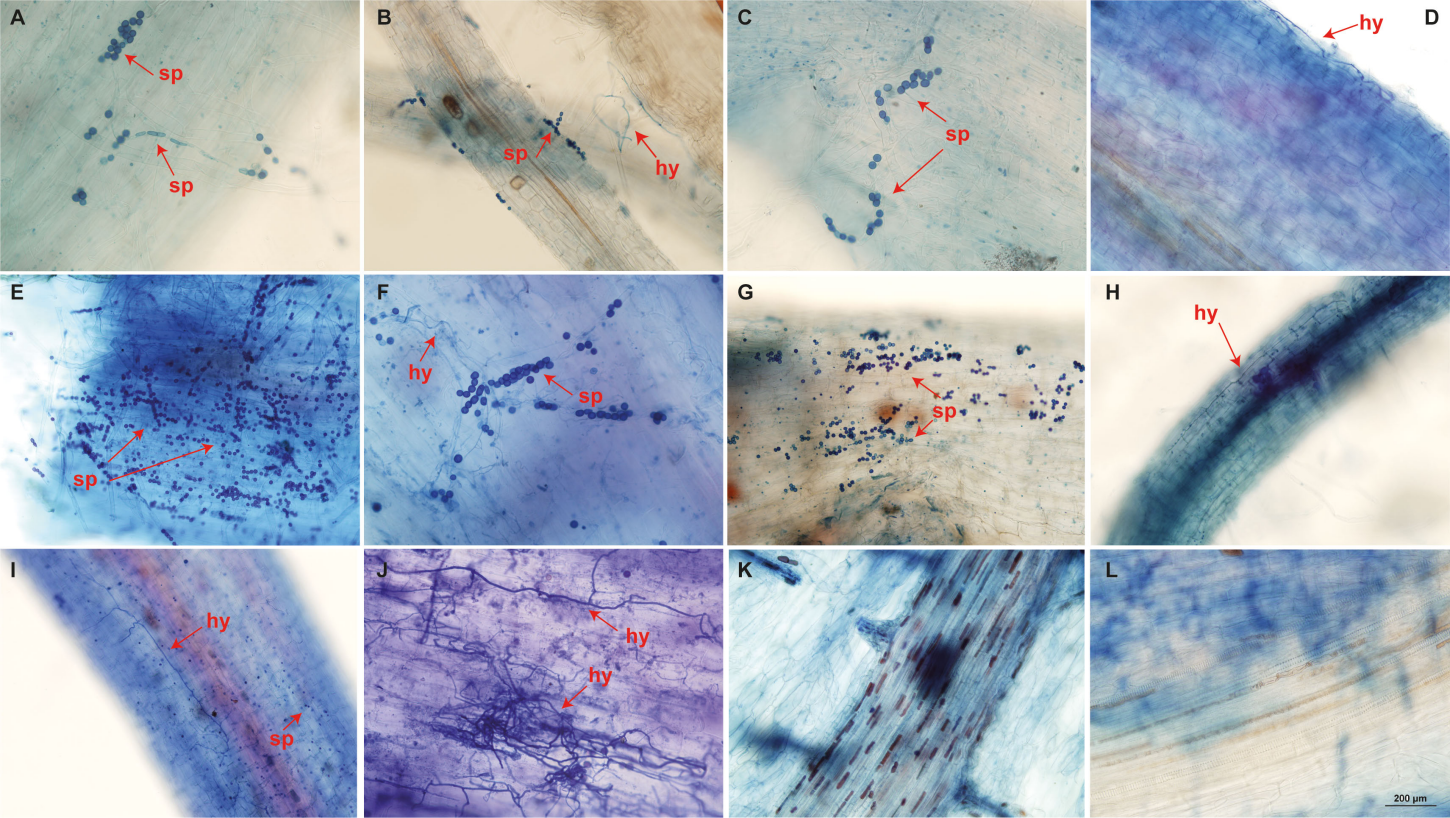
Root clearing of ‘Calcutta 4’ **(A-E)** and ‘Prata-anã’ **(F-J)** at 1, 2, 4, 8, and 15 days after inoculation (DAI), respectively. Arrows indicate Foc STR4 spores (sp) and hyphae (hy). **(K)** non-inoculated C4 control; **(L)** non-inoculated PA control (Bar = 200 µm).

SEM analysis showed only the presence of spores, potentially chlamydospores, on the root surface of ‘Calcutta 4’ at all examined time points (**Figures 3A–C**). In contrast, ‘Prata-anã’ showed spores (**Figures 3D, E**), abundant hyphae and conidiophores on the root surface by 4 DAI (**Figure 3F**). No fungal-related structures were observed in the non-inoculated controls of either genotype (**Figures 3G, H**). Clearing and staining of root samples revealed spore germination, hyphal growth, and chlamydospore accumulation exclusively on the outer root surface of ‘Calcutta 4’ at all examined time points (**Figures 4A–E**), with no evidence of colonization within internal root tissues. Similarly, spores, hyphae and chlamydospores were abundant on the outer root surface of ‘Prata-anã’ at 1 and 2 DAI (**Figures 4F, G**). In contrast, a clear colonization of internal root tissues by Foc STR4 was evident only in ‘Prata-anã’, particularly at 4, 8, and 15 DAI (**Figures 4H–J**). Non-inoculated controls of both genotypes exhibited an absence of fungal spores or hyphae (**Figures 4K, L**).

### 3.2 Fusarium wilt symptoms in ‘Calcutta 4’ and ‘Prata-anã’

**Figure 5:**
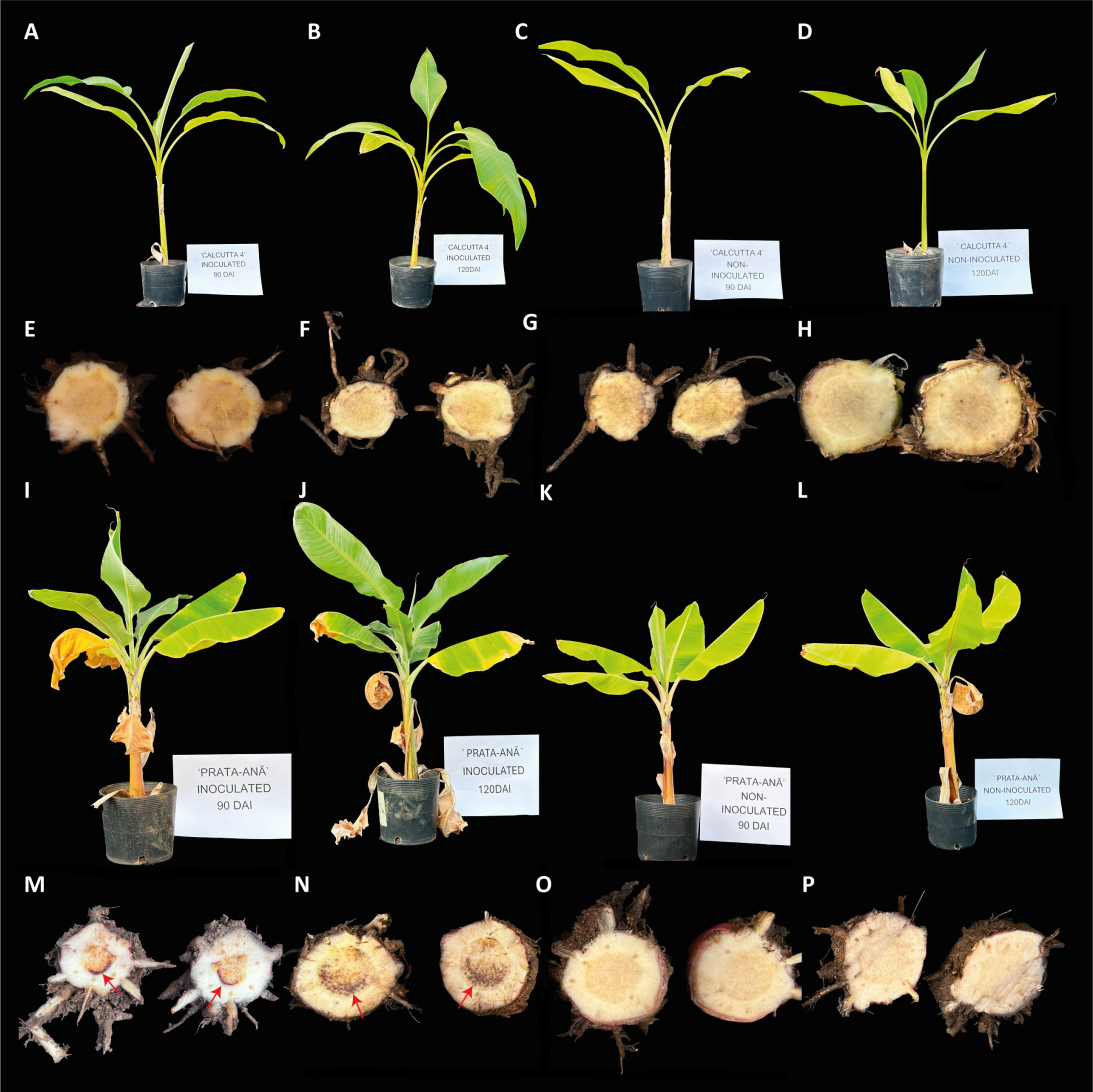
Fusarium Wilt symptoms in contrasting cultivars ‘Calcutta 4’ (resistant) and ‘Prata-anã’ (susceptible) to *Fusarium oxysporum* f. sp. *cubense* race STR4 at 90 and 120 DAI (days after inoculation). **(A-D)** external symptoms in ‘Calcutta 4’: **(A)** 90 DAI, **(B)** 120 DAI, **(C)** control at 90 DAI, **(D)** control at 120 DAI, **(E, H)** internal symptoms in ‘Calcutta 4’ rhizome, **(E)** 90 DAI, **(F)** 120 DAI, **(G)** control at 90 DAI, **(H)** control at 120 DAI. **(I-L)** external symptoms in ‘Prata-anã’. **(I)** 90 DAI, **(J)** 120 DAI, **(K)** control at 90 DAI, **(L)** control at 120 DAI, **(M-P)** internal symptoms in ‘Prata-anã’ rhizome, **(M)** 90 DAI, **(N)** 120 DAI, **(O)** control at 90 DAI, **(P)** control at 120 DAI. Arrows indicate the presence of necrosis symptoms.

Fusarium wilt symptoms were assessed at 90 and 120 DAI in both inoculated and control plants of resistant ‘Calcutta 4’ and susceptible ‘Prata-anã’ (**Figure 5**). In the case of ‘Calcutta 4’ plants, no Fusarium wilt symptoms were observed in either inoculated or non-inoculated control plants at either time point (**Figures 5A–H**). In contrast, inoculated ‘Prata-anã’ plants exhibited characteristic wilt symptoms at both 90 and 120 DAI, including above ground symptoms of yellowing of older leaves progressing to younger leaves (**Figures 5I, J**), and necrosis of rhizome tissues (**Figures 5M, N**). Non-inoculated control plants of ‘Prata-anã’ (**Figures 5K, L, O, P**) did not display any disease symptoms.

### 3.3 RNA-seq data overview in ‘Calcutta 4’

Illumina NovaSeq 6000 PE100 sequencing resulted in 447,843,971 reads from the cDNA libraries representing the treatments 1, 2, and 4 DAI with *Foc* STR4 218A and non-inoculated controls in ‘Calcutta 4’. Following filtering, the total number of reads was reduced to 437,740,123, with 326,407,220 originating from the inoculated treatments and 111,332,903 from the non-inoculated controls. Between 77% and 92% of the ‘Calcutta 4’ reads were correctly mapped to 36,769 gene models in the *M. acuminata* subsp. *malaccensis* DH-Pahang v.4 reference genome (**Table 2**). Illumina RNA-Seq raw sequence data were deposited in the NCBI Sequence Read Archive (SRA) database as a BioProject, accessible under SRA submission number SUB14923683 and BioProject accession number PRJNA1198017.

**Table 2:** Statistics following mRNA sequencing and initial analysis of sequence reads from the interaction between *Musa acuminata* subsp. *burmannica* accession ‘Calcutta 4’ and *Fusarium oxysporum* f. sp. *cubense* STR4.

Organism	Treatment	*Reads* sequenced	*Reads* Trimmomatic	Reads Mapped (%)
*M. acuminata* subsp. *burmannica* accession ‘Calcutta 4’	1DAI	42.533.774	41.569.176	85,24%
	1DAI	38.180.151	37.335.221	85,10%
	1DAI	31.101.530	30.430.312	85,40%
	2DAI	34.312.404	33.473.876	90,73%
	2DAI	35.681.837	34.866.391	89,86%
	2DAI	40.301.477	39.464.423	77,82%
	4DAI	46.284.431	45.209.609	90,66%
	4DAI	33.534.412	32.867.273	91,13%
	4DAI	31.879.599	31.190.939	90,29%
	Control 1DAI	34.727.260	33.867.962	91,24%
	Control 2DAI	35.592.084	34.707.254	84,81%
	Control 4DAI	43.715.012	42.757.687	88,40%

### 3.4 Differential gene expression analysis in ‘Calcutta 4’

**Figure 6:**
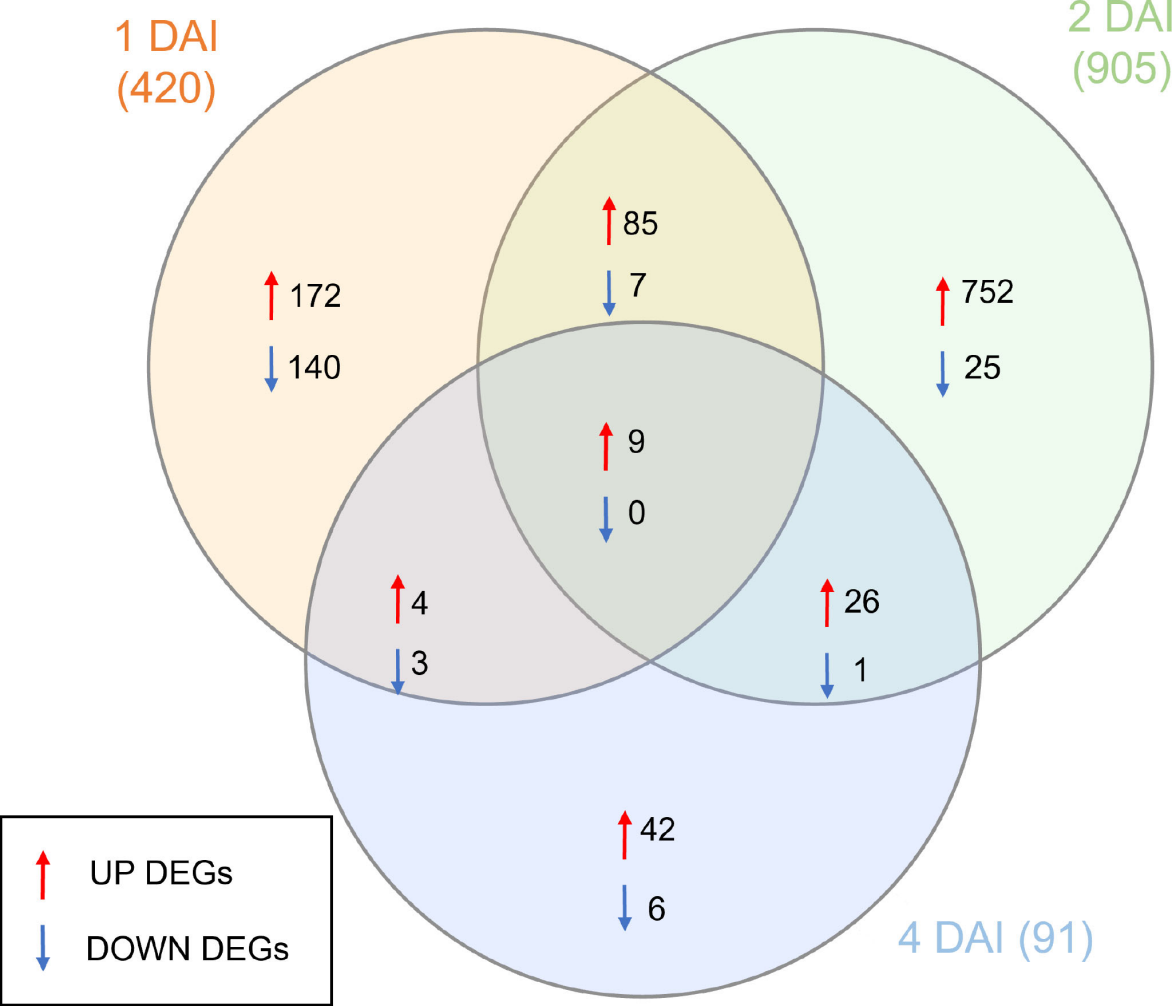
Venn diagram summarizing the numbers of differentially expressed genes (DEGs) observed in RNA-seq data in *Musa acuminata* subsp. *burmannica* accession ‘Calcutta 4’ at 1, 2, and 4 days after inoculation (DAI) with *Fusarium oxysporum* f. sp. *cubense* STR4. Red arrows indicate positively regulated genes, while blue arrows indicate negatively regulated genes. DEGs were considered significant when relative gene expression between an inoculated and corresponding non-inoculated control treatment showed at least a ≥2-fold FC and considering a false discovery rate (FDR)-adjusted P-value (padj) of p ≤0.05. Overlapping regions indicate DEGs common to different time points.

**Figure 7:**
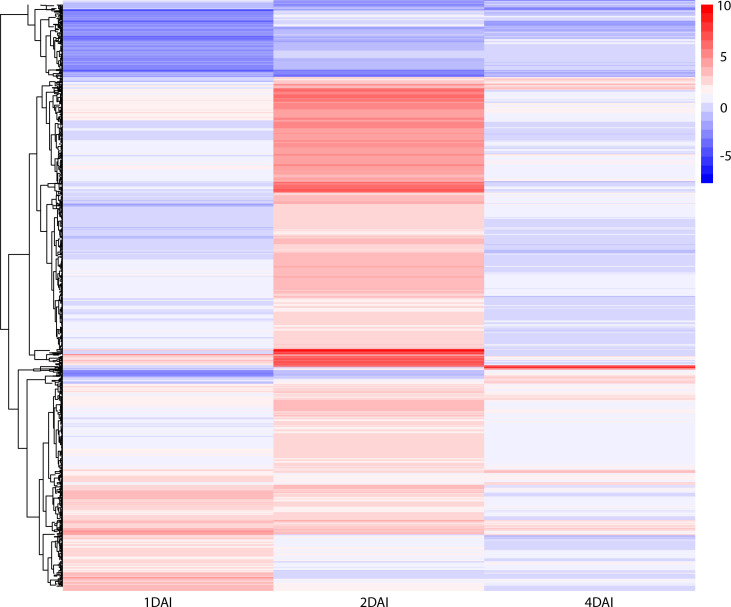
Heatmap of differentially expressed genes in *Musa acuminata* subsp. *burmannica* accession ‘Calcutta 4’ at 1, 2, and 4 days after inoculation (DAI) with *Fusarium oxysporum* f. sp. *cubense* subtropical race 4. The red color indicates positive regulation while the blue color indicates a negative regulation of DEGs in inoculated treatments in relation to non-inoculated controls.

DEGs with significant fold change (at least ≥2-fold and at a probability level of p ≤ 0.05) between inoculated and non-inoculated control treatments were identified following comparison of mapped read counts. A total of 1,416 DEGs were identified in total across the three examined time points (**Supplementary Table S1**). Of these, 1,223 were upregulated, with 270, 872, and 81 DEGs at 1, 2, and 4 DAI, respectively. Conversely, 193 DEGs were downregulated, with 150, 33, and 10 identified at 1, 2, and 4 DAI, respectively (**Figure 6**). The greatest number of DEGs was observed at 2 DAI, with 905, followed by 1 DAI with 420, and 4 DAI with 91 DEGs. Comparison of DEGs across treatments revealed a greater number of shared DEGs at 1 DAI and 2 DAI (**Figure 6**), indicating a higher expression of certain genes in the early stages of *Foc* STR4 infection in ‘Calcutta 4’. Changes in expression of shared DEGs across treatments are shown in **Figure 7**, where shades of red represent upregulation, and shades of blue represent downregulation. Most genes were highly expressed at 2 DAI, with a distinct expression pattern observed at 1 and 4 DAI. These data highlight the significance of the 2 DAI time point in modulating the early defense response of ‘Calcutta 4’ to *Foc* STR4 (**Figure 7**).

### 3.5 Functional analysis of DEGs in ‘Calcutta 4’

#### 3.5.1 Gene ontology

**Figure 8:**
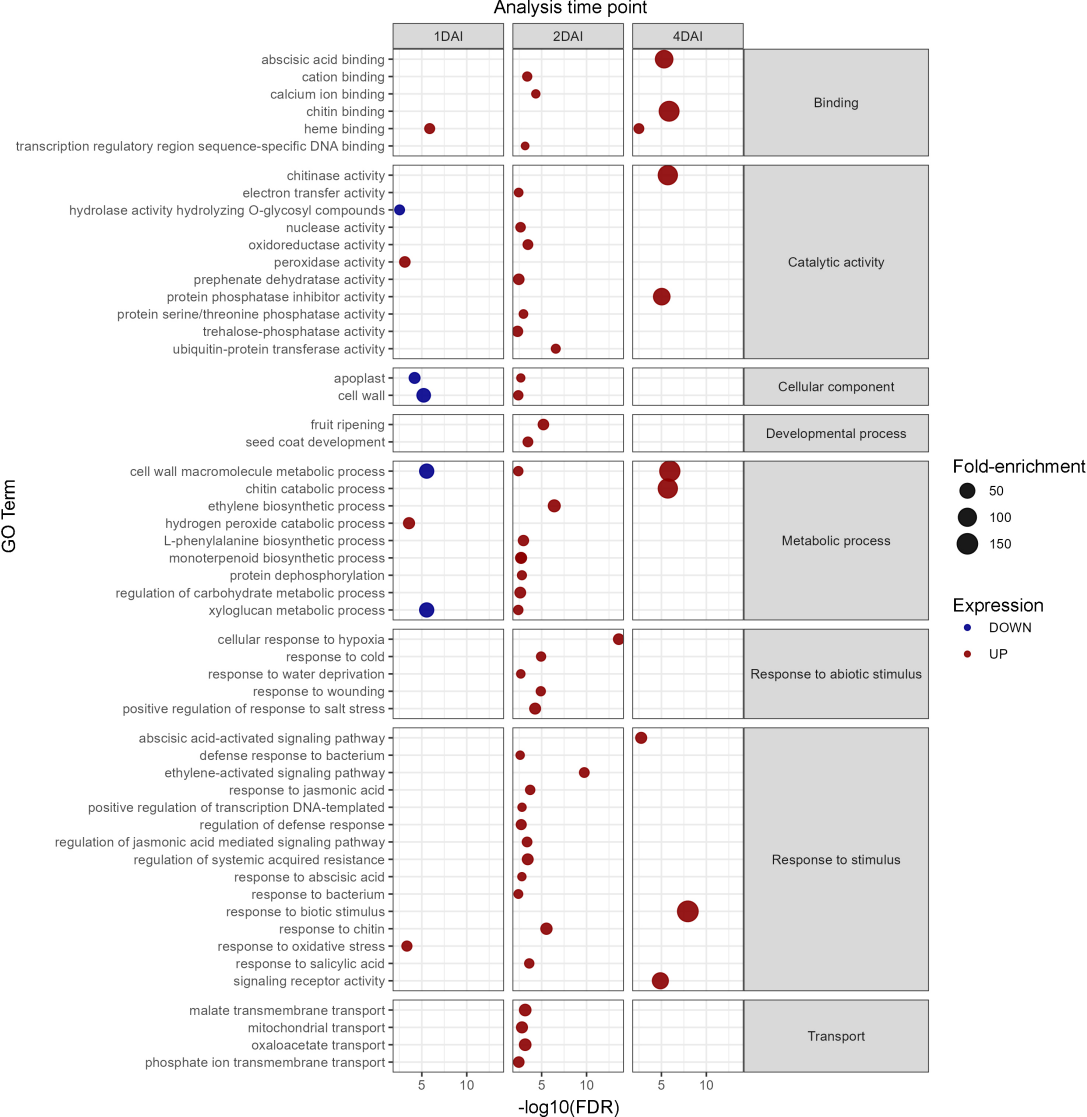
Gene ontology analysis of differentially expressed genes (DEGs) in *Musa acuminata* subsp. *burmannica* accession ‘Calcutta 4’, both positively and negatively regulated at 1, 2, and 4 days after inoculation (DAI).

GO analysis revealed that the upregulated DEGs were enriched in 81 biological processes, seven molecular functions, and 40 cellular component categories, with most terms related to the 2 DAI treatment. Downregulated DEGs were enriched in 20 biological processes, three molecular functions, and 14 cellular component categories, again mostly associated with the 2 DAI treatment, with no enrichment observed for the 4 DAI treatment. Key processes related to biotic stress were identified among the upregulated DEGs, including pathways associated with early pathogen recognition, chitin response, oxidative stress, membrane receptor signaling, chitin binding, and several signaling pathways related to plant hormones such as ethylene, jasmonic acid (JA), and abscisic acid (ABA) (**Figure 8**). Differential modulation of cellular components, particularly related to cell wall and apoplast modifications were observed between 1 and 2 DAI, potentially indicating structural alterations, such as callose deposition, hindering pathogen colonization of host tissues. Negative regulation of secondary root formation, which is the primary entry point for *Foc* STR4 in banana roots, was also observed at 2 DAI.

#### 3.5.2 KEGG pathway analysis

At 1 DAI and 2 DAI, upregulated DEGs were involved in MAPK signaling pathways related to early responses to pathogen attack, cell death, ROS, hydrogen peroxide production involved in programmed cell death, and signaling of ethylene and ABA (**Supplementary Figure S1**). The plant hormone signal transduction pathway map also showed that DEGs, particularly at 2 DAI, were involved in the activation of cell expansion and elongation, ubiquitin-mediated proteolysis, and disease resistance via auxin, ABA, ethylene, brassinosteroid, JA, and SA pathways (**Supplementary Figure S2**). In the plant-pathogen interaction map (**Supplementary Figure S3**), several upregulated DEGs were observed that are involved in the initial recognition of pathogen-associated molecular patterns (PAMPs), associated with PAMP-triggered immunity (PTI). At 1 DAI, WRKY transcription factors (TFs) were observed that typically activate PTI and defense-related genes, leading to the production and accumulation of phytoalexins. A similar pattern was observed at 2 DAI, with enhanced MAPK signaling and the activation of *Pti5*, a transcriptional activator of pathogenesis-related genes. This TF activation likely induced expression of defense-related genes by upregulating PR1 proteins at both 1 and 2 DAI. At 2 DAI, genes involved in effector-triggered immunity (ETI) were observed, such as NLRs *RIN4* and *RPS2*, which play a central role in plant resistance following pathogen infection (**Supplementary Figure S3**).

#### 3.5.3 KOG annotation

**Figure 9:**
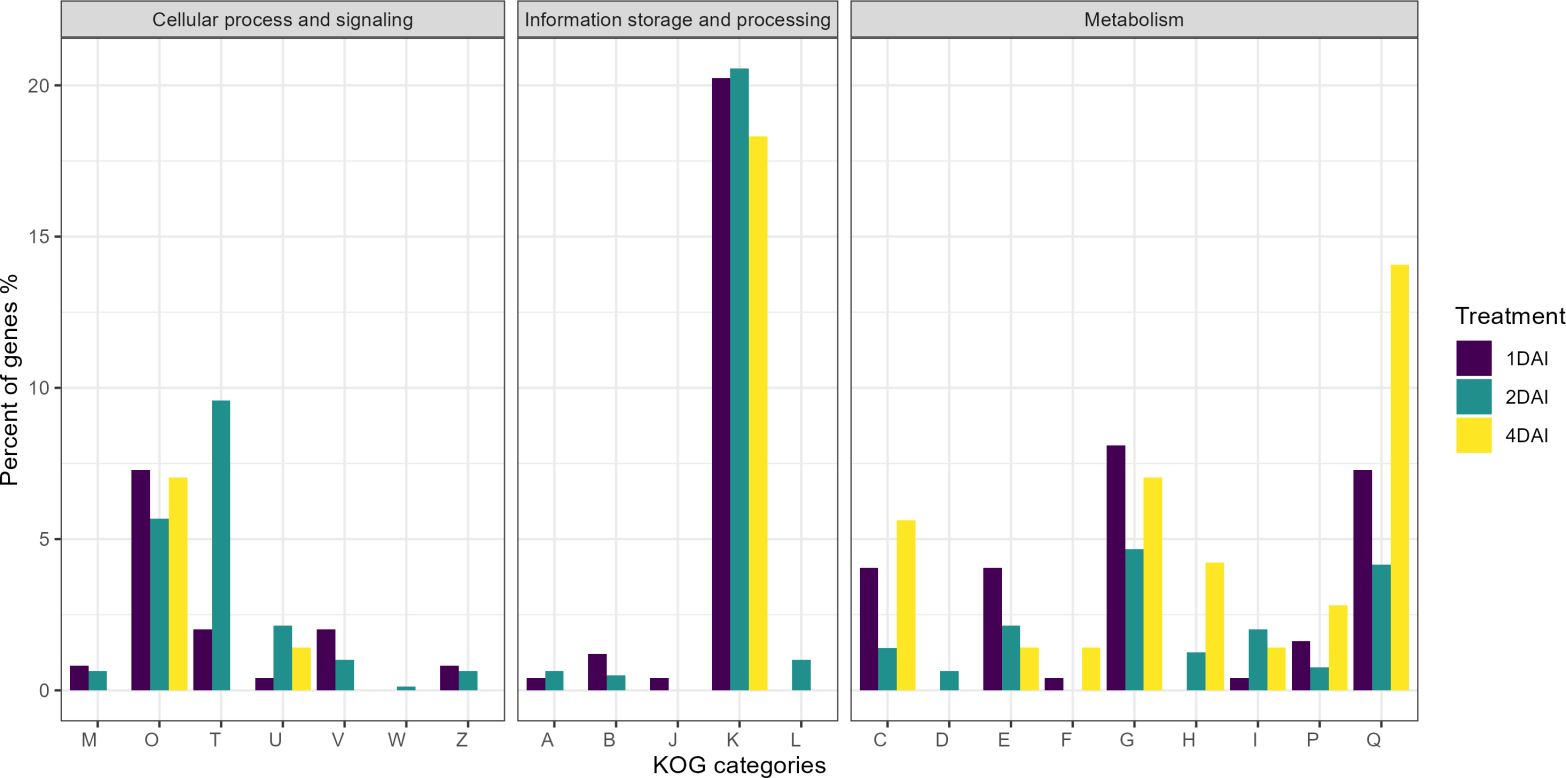
Classification of genes by eukaryotic orthologous groups (KOG) of the differentially expressed genes (DEGs) that are positively regulated in *Musa acuminata* subsp. *burmannica* accession ‘Calcutta 4’ after infection with *Fusarium oxysporum* f. sp. *cubense* STR4, at 1, 2, and 4 days after inoculation. KOG categories: **(A)** RNA processing and modification, **(B)** Chromatin structure and dynamics, **(C)** Energy production and conversion, **(D)** Cell cycle control and mitosis, **(E)** Amino acid metabolism and transport, **(F)** Nucleotide metabolism and transport, **(G)** Carbohydrate metabolism and transport, **(H)** Coenzyme metabolism and transport, **(I)** Lipid metabolism and transport, **(J)** Translation, ribosomal structure and biogenesis, **(K)** Transcription, **(L)** Replication, recombination and repair, **(M)** Cell wall/membrane/envelope biogenesis, **(O)** Post-translational modification, protein turnover, chaperones, **(P)** Inorganic transport and metabolism, **(Q)** Secondary metabolities biosynthesis, transport and catabolism, **(T)** Signal transduction mechanisms, **(U)** Intracellular trafficking, secretion and vesicular transport, **(V)** Defense mechanisms, **(W)** Extracellular structures, **(Z)** Cytoskeleton.

Of the 1,416 DEGs, 1,177 were functionally predicted and annotated in the KOG database, with 233, 712, and 67 upregulated at 1, 2, and 4 DAI, respectively, and 131, 25, and 9 downregulated at 1, 2, and 4 DAI, respectively (**Figure 9**). Of the 15 DEGs in the KOG V category (defense mechanisms), five were upregulated at 1 DAI, namely *Macma4_05_g11220* (Abhydrolase3 domain-containing protein), *Macma4_05_g23430*, *Macma4_08_g11580*, *Macma4_11_g03200* (Protein DETOXIFICATION), and *Macma4_09_g11990* (putative Protein DETOXIFICATION 40). Two DEGs were downregulated at 1 DAI: *Macma4_03_g03150* (3Beta_HSD domain-containing protein) and *Macma4_07_g04620* (Protein DETOXIFICATION). The remaining eight DEGs were upregulated at 2 DAI, comprising *Macma4_02_g12060* (conserved hypothetical protein), *Macma4_02_g13270*, *Macma4_08_g28030* (Protein DETOXIFICATION), *Macma4_06_g14580* (1-aminocyclopropane-1-carboxylate oxidase), *Macma4_06_g27140* (3Beta_HSD domain-containing protein), *Macma4_06_g29370* (putative Phosphatidylglycerophosphate phosphatase PTPMT1), *Macma4_06_g32160*, and *Macma4_08_g05580* (Abhydrolase_3 domain-containing protein).

#### 3.5.4 Mapman metabolic pathway analysis

**Figure 10:**
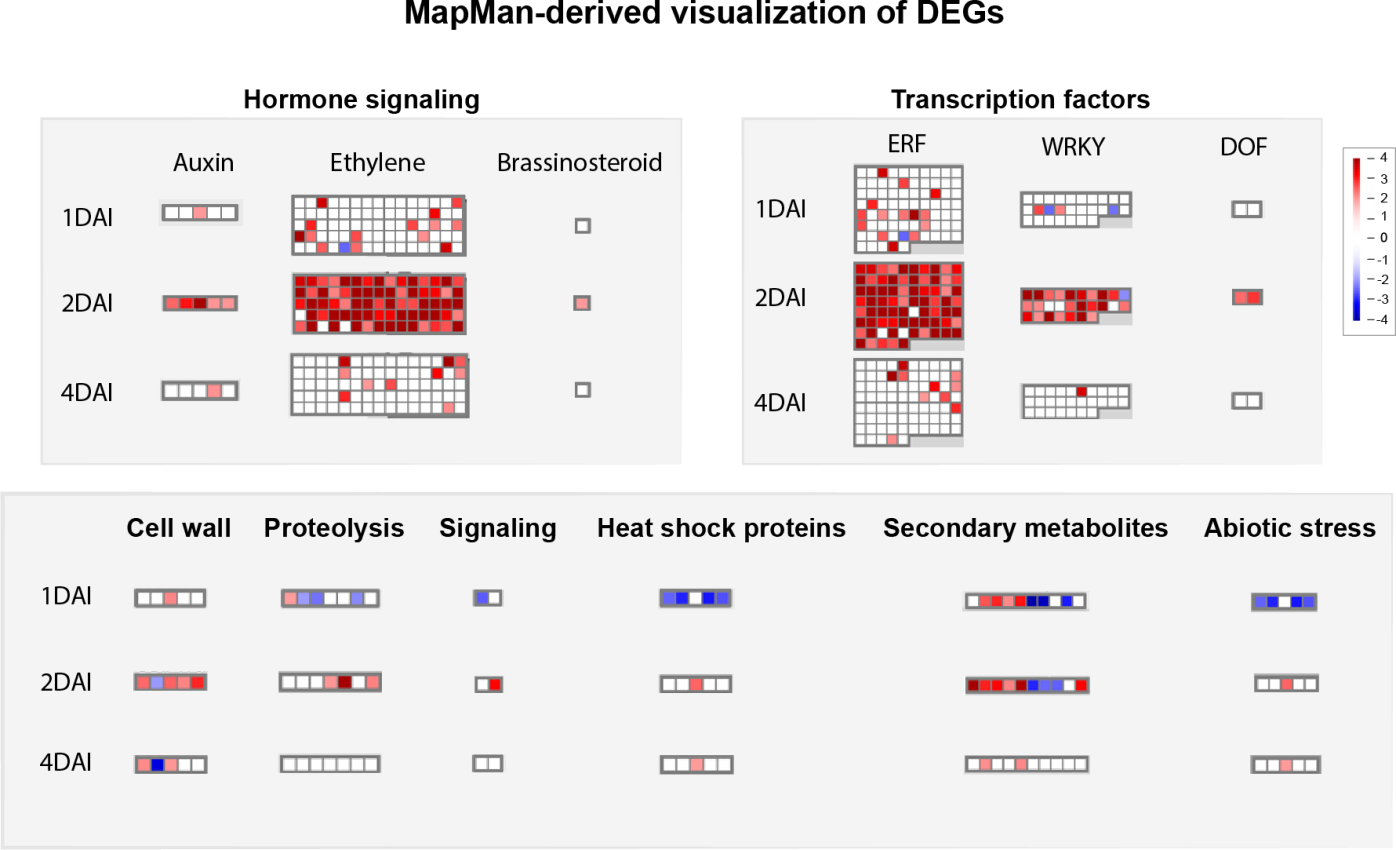
Mapman-based functional prediction mapping of differentially expressed genes (DEGs) in *Musa acuminata* subsp. *burmannica* accession ‘Calcutta 4’ after infection with *Fusarium oxysporum* f. sp. *cubense* STR4, at 1, 2, and 4 days after inoculation.

Functional annotation through MapMan analysis revealed differential modulation of genes involved in key signaling pathways and defense mechanisms in ‘Calcutta 4’ in response to *Foc* STR4 infection over the time course investigated. Of the 1,416 DEGs, 1,370 functional categories (bins) were generated, with 231 related to regulation, 219 to biotic stress, 142 to transcription, 44 to metabolism, 32 to cellular response, and 13 to secondary metabolism. Overall, signaling pathways involving plant hormones, TFs, and secondary metabolites were activated by a significant number of upregulated DEGs at 2 DAI in ‘Calcutta 4’, compared to 1 and 4 DAI (**Figure 10**). These DEGs, with Log2FC values ranging from 2 to 4 at 2 DAI, were responsible for auxin, ethylene, and brassinosteroid signaling, activation of TFs such as ERF (ethylene response factor), WRKY (which regulates transcription by activating a signaling network in response to biotic and abiotic stress), and DOF (DNA-binding with one finger), which plays a key role in plant growth, development, and stress responses. Moreover, secondary metabolite pathways were activated at 1 and 2 DAI, indicating that these compounds were produced following the initial pathogen attack by STR4. Other pathways, such as cell wall, proteolysis, signaling, and abiotic stress-related pathways, also showed greater activation at 2 DAI (**Figure 10**).

### 3.6 Validation of *in silico* data via RT-qPCR

**Figure 11:**
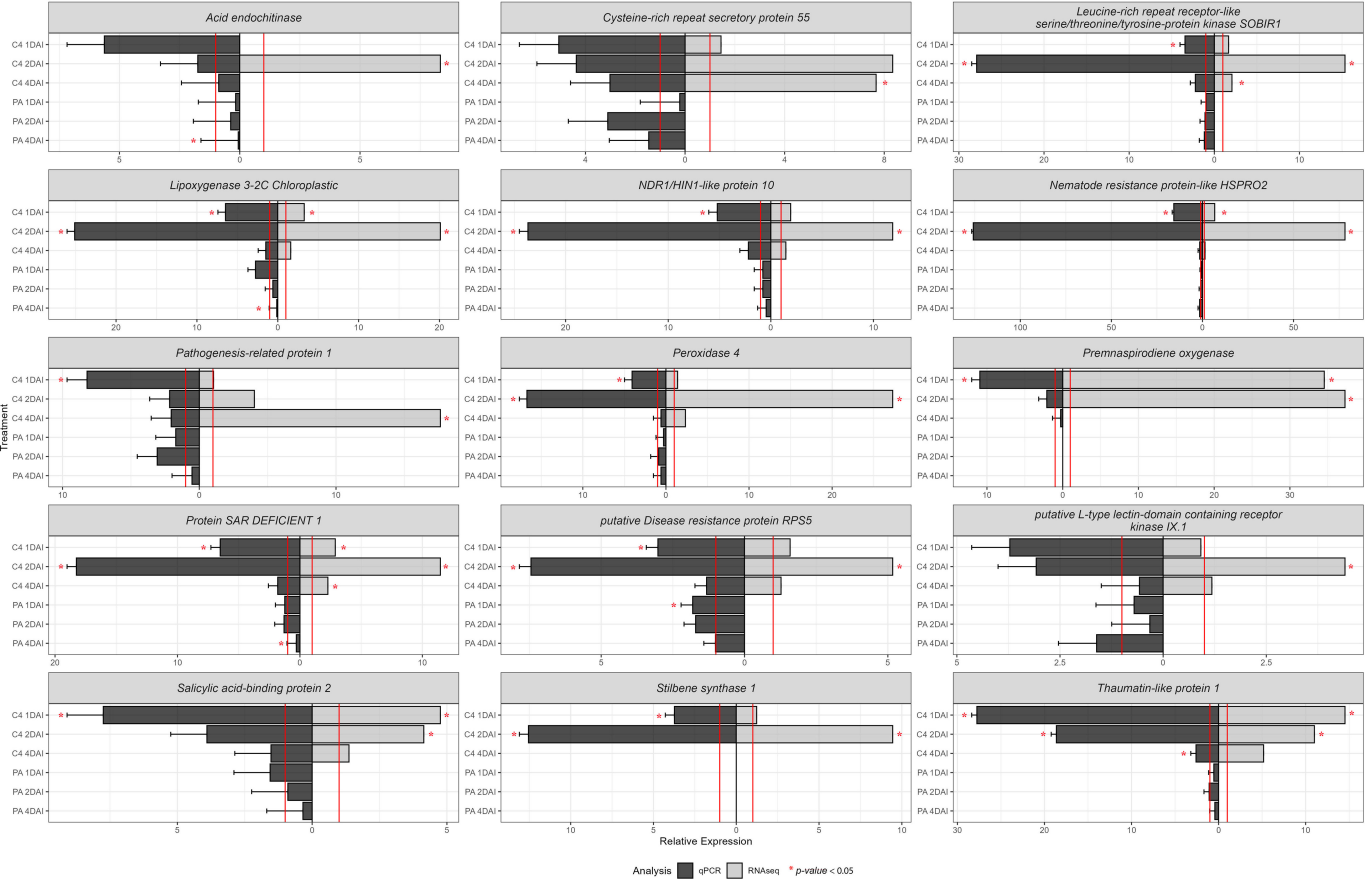
RT-qPCR analysis of relative expression of 15 differentially expressed upregulated genes identified following RNA-seq analysis of the interaction between *Musa acuminata* subsp. *burmannica* accession ‘Calcutta 4’ and *Fusarium oxysporum* f. sp. *cubense* STR4, at 1, 2, and 4 days after inoculation. RT-qPCR data was analysed using the *2 ^-ΔΔCT^* method (Livak and Schmittgen, 2001). The expression of the control treatments is equal to 1 and marked by a red line. Both values for RT-qPCR and RNA-seq were positive.

Validation of *in silico*-derived differential gene expression data for ‘Calcutta 4’ and equivalent genes in ‘Prata-anã’ was conducted by RT-qPCR, with expression compared in inoculated samples relative to non-inoculated controls (**Figure 11**) (**Supplementary Table S2**). A total of 15 genes were analyzed from up-regulated DEGs in ‘Calcutta 4’ RNA-seq data, following selection based on their potential involvement in the upstream to downstream immune response. All examined genes for ‘Calcutta 4’ displayed relative expression trends similar to those obtained by RNA-seq (**Figure 11**).

Thaumatin-like protein 1 (*Macma4_05_g17130*) exhibited a significant relative expression at 1, 2, and 4 DAI in ‘Calcutta 4’ in comparison with the non-inoculated control. Overexpression of the genes Leucine-rich repeat receptor-like serine/threonine/tyrosine-protein kinase SOBIR1 (*Macma4_02_g09450*), Lipoxygenase 3-2C Chloroplastic (*Macma4_03_g08040*), putative Disease resistance protein RPS5 *(Macma4_04_g37480*), Peroxidase 4 (*Macma4_06_g16370*), NDR1/HIN1-like protein 10 (*Macma4_09_g31980*), Nematode resistance protein-like HSPRO2 (*Macma4_10_g08320*), Stilbene synthase 1 (*Macma4_10_g24620*), and Protein SAR DEFICIENT 1 (*Macma4_08_g16850*) was significant at 1 and 2 DAI in ‘Calcutta 4’. For the genes Salicylic acid-binding protein 2 (*Macma4_04_g03250*), Premnaspirodiene oxygenase (*Macma4_07_g21750)*, and Pathogenesis-related protein 1 (*Macma4_03_g08420*), significant overexpression occurred only at 1 DAI, although positive trends were also observed in other treatments, aligning with RNA-seq data. In contrast to data for ‘Calcutta 4’, where both RNA-seq and RT-qPCR confirmed upregulation of gene expression at 1, 2 and 4 DAI, Stilbene synthase 1 (*Macma4_10_g24620*) and Premnaspirodiene oxygenase (*Macma4_07_g21750*), which are involved in phytoalexin biosynthesis, showed no relative expression data by RT-qPCR in any treatments in ‘Prata-anã’. Other genes, including Acid endochitinase (*Macma4_01_g21300*), Leucine-rich repeat receptor-like serine/threonine/tyrosine-protein kinase SOBIR1 *(Macma4_02_g09450*), Lipoxygenase 3-2C Chloroplastic (*Macma4_03_g08040*), NDR1/HIN1-like protein 10 (*Macma4_09_g31980*), Nematode resistance protein-like HSPRO2 (*Macma4_10_g08320*), Pathogenesis-related protein 1 (*Macma4_03_g08420*), Peroxidase 4 *(Macma4_06_g16370*), Protein SAR DEFICIENT 1 (*Macma4_08_g16850*), putative Disease resistance protein RPS5 (*Macma4_04_g37480*), putative L-type lectin-domain containing receptor kinase IX.1 (*Macma4_06_g30880*), Salicylic acid-binding protein 2 (*Macma4_04_g03250*), and Thaumatin-like protein 1 (*Macma4_05_g17130*), also showed higher relative expression in ‘Calcutta 4’ than in ‘Prata-anã’.

These results indicate a trend towards increased expression of genes associated with resistance to *Foc* in ‘Calcutta 4’, especially in the early stages of the interaction at 2 DAI, contrasting to an absence of upregulation of expression in the susceptible genotype ‘Prata-anã’. **Figure 11** also highlights expression trends observed through RT-qPCR analysis supporting those observed by *in silico* RNA-seq analysis.

## 4 Discussion

Bananas are among the world’s most important fruits, with approximately 80% of production consumed domestically and 20% destined for global export markets. With regard to the latter, 90% of production occurs in Central America, South America, and the Philippines (FAO, 2024). Further expansion of banana cultivation is limited as a result of certain abiotic and biotic stresses, with the greatest threat to production today imposed by *Foc*. Whilst *Foc* Race 1 devastated global production of the ‘Gros Michel’ variety in the middle of the 20^th^ Century, the emergence of Race 4 is now threatening the ‘Gros Michel’ replacement cultivars within the ‘Cavendish’ subgroup, with around 80% of the cultivars grown globally considered susceptible to *Foc* TR4 (Bermúdez-Caraballoso et al., 2020). Losses are considerable, particularly across much of Asia, Australia, Mozambique, Oman, Turkey, Venezuela, Colombia, and Peru, where *Foc* TR4 is present. Entry of *Foc* TR4 into Brazil also now appears to be only a matter of time, as is the case for other banana-producing nations in the Americas (Ploetz et al., 2015; Munhoz et al., 2024). In addition to *Foc* TR4, STR4 is also widely disseminated and now present in China, Australia, Taiwan, South Africa, Morocco, and Brazil (Munhoz et al., 2024).

Given the limited genetic diversity in commercial *Musa* cultivars and the constraints to crop improvement in conventional *Musa* breeding programs, research efforts have focused on candidate resistance gene discovery via transcriptome analysis of the interaction between resistant genotypes and diverse pathogens (Castañeda et al., 2017; Tripathi et al., 2019; Pinheiro et al., 2022; Lantican et al., 2023; Anuradha et al., 2024).

Given the significant threat posed by *Foc* TR4, a greater number of transcriptomic studies on *Foc* have to date focused on this pathogen race. While some susceptible *Musa* genotypes show early activation of immune pathways, their responses are often insufficient to halt disease progression. In contrast, resistant cultivars and wild relatives exhibit more robust and coordinated defense mechanisms, often involving both innate immunity and complex downstream signaling.

### 4.1 Defense responses in susceptible cultivars

Wang et al. (2012) investigated the compatible response in Cavendish subgroup ‘Brazilian’ to TR4, demonstrating activation of pathogen recognition and defense signaling pathways (such as SA, JA, and ET) at 1- and 2-days post-inoculation, with PR protein and lignification enzyme expression at 4 DAI. Early-stage overexpression of peroxidases also suggested ROS activation after TR4 infection. However, these responses were not durable or strong enough to prevent disease establishment.

A comparative study in the Cavendish cultivar ‘Baxi’, resistant to *Foc* R1 and susceptible to *Foc* TR4, revealed similar expression patterns at 2 DAI to both races. A notable exception was the suppression of an *allene oxide synthase* gene during *Foc* TR4 infection, suggesting that TR4 infection may reduce JA-mediated defense (Li et al., 2013). In a similar study with the Cavendish cultivar ‘Brazilian’ (resistant to *Foc* R1, susceptible to *Foc* TR4), *Foc* R1 induced expression changes in RLK receptors, TFs, metabolic pathways, lignin/flavonoid synthesis, PR-1 and PR-4 chitinase genes, and secondary metabolites, including thos related to glucosinolates, alkaloids, and terpenoids (Dong et al., 2020). In contrast, gene expression and pathway changes after *Foc* TR4 infection were either unaffected or only weakly affected, highlighting contrasting early-stage responses to each *Foc* race. Li et al. (2023) further demonstrated suppression of SA pathways during early *Foc* TR4 infection in the *Foc* TR4-susceptible cultivar ‘Brazilian’ when compared to *Foc* R1 infection, to which the genotype is resistant. Notably, RIN4, an NLR protein involved in resistance via the membrane receptor RPM1, was overexpressed only after *Foc* R1 infection, possibly explaining the susceptibility of ‘Brazilian’ to *Foc* TR4.

### 4.2 Enhanced defense activation in resistant cultivars and mutants

In contrast to susceptible genotypes, resistant cultivars and mutants exhibit a more coordinated and amplified transcriptional response. Studies on the resistant ‘Cavendish’ mutant ‘Nongke No1’ identified nine major immunity-related pathways activated in response to *Foc* TR4, comprising PAMP perception via PRRs, ETI, ion flux changes, TFs, oxidative burst, PRs, programmed cell death, plant hormones, and cell wall modification (Li et al., 2012). Enhanced signaling through SA and JA pathways, along with glycolysis and glyoxylate cycle-related DEGs, were also observed in this resistant genotype when compared to expression in susceptible ‘Baxi’ (Li et al., 2019b). In TR4-resistant ‘Yueyoukang 1’, genes associated with pathogen recognition (CeBiP, BAK1, NLRs), PR proteins, TFs, and antimicrobial activity (glucanases, and endochitinases) were more strongly expressed in comparison to susceptible ‘Brazilian’ following *Foc* TR4 infection (Bai et al., 2013). Further transcriptomic analyses identified strong induction of PR proteins (*PR-3*, *PR-4*) and numerous cell wall-modifying enzymes, including pectinesterases, β-glucosidases, xyloglucan endotransglucosylase/hydrolases, and endoglucanases (Niu et al., 2018), all contributing to cell wall reinforcement and pathogen containment. Similarly, in *Foc* TR4-resistant ‘Guijiao 9’, overexpression of genes encoding the disease resistance protein *RPM1*, the TF *PTI6*, the elicitor receptor *CeBiP*, calcium-dependent protein kinase (*CDPK*), ubiquitin pathway regulators such as *UBE2H*, and zinc-finger proteins (*RCHY1*) was reported at 2–6 DAI compared to the susceptible ‘Williams’ cultivar (Sun et al., 2019). These findings collectively highlight the complex, multi-layered immune network deployed by resistant *Musa* cultivars in response to *Foc* TR4.

### 4.3 Constitutive and induced defense in wild resistant genotypes

In the case of wild species, Zhang et al. (2019) also observed a constitutive defense response in *Foc* TR4-resistant ‘Pahang’, involving WRKY and TIFY TFs, and putative disease resistance genes. Upon TR4 challenge, significant upregulation of genes involved in early defense and antimicrobial activity, including *PR1*, chitinases (*CHIT*), aspartic proteases (*ASP*), lipases (*GLIP*), peroxidases (*POD*), polyphenol oxidases (*PPO*), and cytochrome P450s, was observed. In another comparative study comparing *Foc*-resistant ‘Calcutta 4’ (AA) with the susceptible cultivar ‘Kadali’ (AA), 18 defense-related genes involved in signaling, ROS, lignification, and PR pathways, were identified as playing roles in the defense response in ‘Calcutta 4’ against *Foc* R1 (Kotari et al., 2018).

Our study provides a significant perspective on the wild accession ‘Calcutta 4’, which is resistant to all *Foc* races. Focusing on the interaction with *Foc* STR4, which is genetically the closest race to TR4 and widespread in Brazil, Australia, the Canary Islands, China, South Africa, and Taiwan (Munhoz et al., 2024), this study provides a first transcriptome-level investigation of the resistance response to this widespread race. Histological analyses showed a rapid defense response following *Foc* STR4 challenge, with callose formation and phenolic compound deposition observed at 1 and 2 DAI. Such responses were absent in ‘Prata-anã’ over the examined time course. When comparing *Foc* STR4 colonization in resistant ‘Calcutta 4’ and susceptible ‘Prata-anã’, the presence of chlamydospores and hyphae on the root exterior was similar in both genotypes up to 4 DAI. However, at 8 and 15 DAI, hyphae were more prevalent in ‘Prata-anã’, both externally and internally within root cells, with only chlamydospores observed in ‘Calcutta 4’. Previously, Li et al. (2019b) similarly demonstrated a greater abundance of *Foc* TR4 hyphae and spores in susceptible ‘Baxi’ in comparison to resistant ‘Nongke No.1’ at 27 and 51 hpi. Considering the differences in pathogen growth between these contrasting genotypes, callose and phenolic compounds likely represent defense responses that hinder *Foc* STR4 colonization in ‘Calcutta 4’ root tissues.

**Figure 12:**
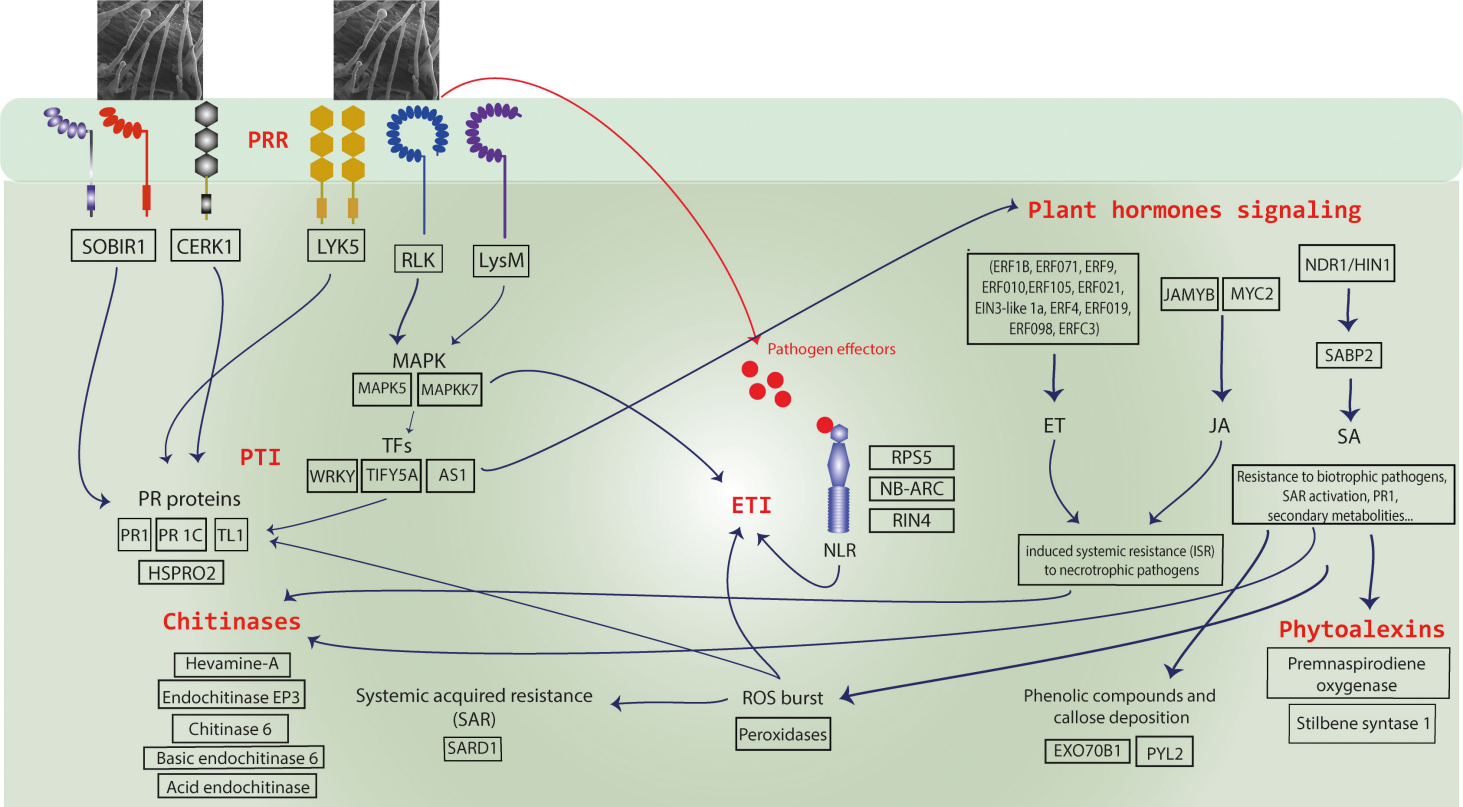
A schematic model of the innate immune responses observed in *Musa acuminata* subsp. *burmannica* accession ‘Calcutta 4’ following infection by F*usarium oxysporum* f. sp. *cubense* subtropical race 4 (STR4). Upregulated candidate resistance genes included PRRs (transmembrane pattern recognition receptors) for recognition of fungal PAMPs (Pathogen associated molecular patterns) and NLR proteins (*R* genes) for recognition of pathogen effectors, triggering Effector-triggered immunity (ETI) and induction of a hypersensitive cell death (HR) response. Downstream defense response-associated genes were also observed amongst upregulated genes, including those encoding pathogenesis-related (PR) proteins, phytohormone pathway-associated proteins, chitinases, proteins involved in systemic acquired resistance (SAR), reactive oxygen species (ROS), and secondary metabolites such as phytoalexins.

Transcriptome data aligned with findings based on histology, with a greater differential expression of genes at 2 DAI. Among the total of 1,416 DEGs identified in RNA-seq analysis, 270 and 872 were overexpressed at 1 and 2 DAI, respectively, with only 81 at 4 DAI. Of these, 752 DEGs were regulated exclusively at 2 DAI, suggesting a rapid response of ‘Calcutta 4’ to the onset of *Foc* STR4 infection, as has been shown in other pathosystems. Seventy-five globally upregulated DEGs were identified as possible agents in the defense response to *Foc* STR4, associated with GO biological processes categorized under response to stimulus. Candidate genes activated in the innate immune response in ‘Calcutta 4’ against *Foc* STR4 infection, associated with PTI and ETI pathways, are summarized in the schematic model in **Figure 12** and listed in **Supplementary Table S3**. Comparison with the literature also reveals that many of the core defense components activated in response to *Foc* STR4 overlap with those triggered by *Foc* TR4, supporting a degree of conservation in the immune response. Key genes and classes common to the resistance response to *Foc* STR4 and *Foc* TR4 are summarized in **Supplementary Table S4**.

### 4.4 PTI responses

Resistance responses to PAMPs involve recognition by PRRs, including RLK and RLP receptors, triggering signaling pathways such as Ca²^+^ influx, ROS production, and MAPK activation (Ding et al., 2022; Ngou et al., 2022). Here, numerous such membrane receptors were modulated during the incompatible response in ‘Calcutta 4’ (**Supplementary Table S3**). These included Protein LYK5 (*Macma4_04_g09660*), a chitin receptor that binds CERK1 (Chitin elicitor receptor kinase 1) (*Macma4_10_g11450*) to form a chitin recognition complex involved in non-host resistance (Cao et al., 2014; Huaping et al., 2017). SOBIR1 (*Macma4_02_g09450*) was also identified, known to activate cytoplasmic signaling, oxidative bursts and MAPK cascades (Gao et al., 2009; Liebrand et al., 2013; Wei et al., 2022). A putative LysM domain receptor-like kinase 4 (*Macma4_04_g01380*) was also identified, implicated in fungal chitin recognition, PRR activation, and PTI. Similarly, Kotari et al., 2018 observed upregulation of PAMP recognition and defense signaling genes in the resistance response of ‘Calcutta 4’ to *Foc* R1, including *CeBiP*, *CERK*-6, *RPK*-2, *LECTIN*, and *MAPKKK-11*. Following PAMP recognition, Thaumatin-like protein 1 (*Macma4_05_g17130*) showed PRR-associated activation at 1 and 2 DAI. Mahdavi et al. (2012) demonstrated a reduced incidence of fusarium wilt caused by TR4 in transgenic ‘Pisang Nangka’ (AAB) bananas expressing a rice Thaumatin-Like Protein gene. Upregulation of a PR-protein 1C (*Macma4_02_g16620*), and a TL1, protein of the PR5 class was also significant in ‘Calcutta 4’ during early interaction with *Foc* STR4, contrasting with the susceptible ‘Prata-anã’ (Dixon et al., 1991; Hakim et al., 2018; Han et al., 2023). Additionally, the nematode resistance protein-like HSPRO2 (*Macma4_05_g32600, Macma4_10_g08320, Macma4_10_g08320, Macma4_11_g23440*) was upregulated at 1 and 2 DAI (**Supplementary Table S3**). Although lacking the canonical nucleotide-binding site, HSPRO2 contains an imperfect LRR domain and contributes to basal resistance against pathogens (Murray et al., 2007; Nemchinov et al., 2017).

### 4.5 Callose deposition

Plants defend against pathogens through various chemical and physical mechanisms. Callose, a β-1,3-glucan polymer, is deposited in response to PRR-triggered signaling following PAMP recognition, contributing to cell wall reinforcement and acting as a matrix for antimicrobial compounds (Luna et al., 2011). In *Arabidopsis*, callose deposition is activated by the flg22 PAMP peptide and regulated by EXO70B1 and EXO70B2 genes (Redditt et al., 2019). Callose deposition can also be influenced by signaling from abscisic acid (ABA) pathways in response to infection by fungi and oomycetes, as demonstrated by the ABA-dependent callose accumulation during *Botrytis cinerea* infection (You et al., 2010). We confirmed through comparative histological analyses in inoculated and non-inoculated ‘Calcutta 4’ and ‘Prata-anã’ that callose deposition occurred exclusively in ‘Calcutta 4’ at 1 and 2 DAI, following interaction with *Foc* STR4. This response coincided with the upregulation of Exocyst complex component EXO70B1 (*Macma4_10_g10790*). Notably, there was also overexpression of the gene Abscisic acid receptor PYL2 (*Macma4_06_g02820)* at 1 and 2 DAI, a regulatory component of the ABA receptor family (García-Andrade et al., 2020), suggesting a role for ABA-mediated signaling in callose deposition in ‘Calcutta 4’ in response to Foc STR4.

### 4.6 Chitinases

Plant chitinases are key components of resistance to biotic stress due to their ability to hydrolyze chitin, a major constituent of fungal cell walls, thus contributing to primary defense against fungal pathogens. In this study, seven chitinases were upregulated in ‘Calcutta 4’ following interaction with *Foc* STR4: Hevamine-A (*Macma4_08_g34260*), Acidic endochitinase (*Macma4_01_g21300*), Chitinase (*Macma4_03_g29600*), Basic endochitinase C (*Macma4_03_g29630*), Chitinase 6 (*Macma4_05_g18850* and *Macma4_05_g18860*), Endochitinase EP3 (*Macma4_06_g23390*), and Chitin-inducible gibberellin-responsive protein 1 (*Macma4_09_g12390*). Chitinase involvement in the defense response has previously been demonstrated in ‘Cavendish’ in response to *Foc* R1 (Dong et al., 2020), in transgenic ‘Gros Michel’ lines expressing a rice chitinase gene (*RCC2*) conferring resistance to black leaf streak disease (Kovács et al., 2013), and in the ‘Furenzhi’ (AA) cultivar engineered with a *Trichoderma harzianum* endochitinase gene (*chit42*) for resistance to *Foc* race 4 (Hu et al., 2013).

### 4.7 Reactive oxygen species and oxidative stress responses

ROS production and SAR activation occur following recognition of pathogen Avr proteins by R proteins. ROS play dual roles in defense: directly reinforcing cell walls to prevent pathogen colonization (Huang et al., 2019) and acting as conserved signaling molecules for chitin perception and MAPK cascades (Kawasaki et al., 2017). In this study, genes associated with oxidative stress responses were predominantly upregulated in ‘Calcutta 4’ at 1 and 2 DAI, including SAR DEFICIENT 1 (SARD1) (*Macma4_08_g16850*) and several peroxidases (**Supplementary Table S3**). Peroxidases are multifunctional stress-responsive enzymes with antioxidant activity and a role in phenolic compound biosynthesis. Prior research on the ‘Calcutta 4’ - *Foc* race 1 interaction showed higher peroxidase levels in this wild subspecies compared to the susceptible ‘Kadali’ (Swarupa et al., 2013).

### 4.8 Effector triggered immunity

ETI is activated through the direct or indirect recognition of race-specific pathogen effectors by NLR R proteins, often resulting in a hypersensitivity response (HR) and localized cell death (Jones and Dangl, 2006). Previously, 52 partial NLR genes were identified in ‘Calcutta 4’ (Miller et al., 2008). In ‘Pahang’, *RGA2* (resistance gene analogue 2) and four additional putative resistance genes were highly expressed upon *Foc* TR4 infection (Zhang et al., 2019a). Using the *RGA2* gene, Dale et al. (2017) developed the first *Foc* TR4-resistant transgenic ‘Cavendish’ cultivar (Harding et al., 2025). In this study, we observed upregulation of an *NDR1/HIN1-like protein 10* gene, involved in CC-NBS-LRR-type NLR signaling (*Macma4_ 09_g31980*), as well as several NLR genes at 2 DAI, including NB-ARC domain-containing proteins (*Macma4_01_g04350, Macma4_01_g04360, Macma4_01_g04380*) and RPS5(*Macma4_04_g37480*).

### 4.9 Transcription factors

TFs are central to signaling and regulation of plant defense pathways under biotic stress. Of the upregulated TFs in ‘Calcutta 4’ during interaction with STR4, most belonged to the TIFY, WRKY, MYB, and MYC families. Two critical WRKY genes were identified: WRKY WRKY28 (*Macma4_11_g08360*) and WRKY WRKY51 (*Macma4_09_g24850*). WRKY51 is involved in NPR1-mediated signaling, essential for SA-induced PR gene expression and pathogen resistance (Ding et al., 2018). WRKY50 and WRKY51 proteins also mediate SA- and low-dependent repression of JA signaling (Gao et al., 2011). TIFY family TFs are plant-specific regulators involved in development and responses to both biotic and abiotic stresses. JAZ proteins, a subfamily of TIFY, function as JA signaling repressors and co-receptors, interacting with *MYC2*, a positive regulator of JA-responsive gene expression (Zhang et al., 2020). In this study, notable TIFY family members were identified, including putative TIFY 5A (*Macma4_02_g16390, Macma4_06_g34540, Macma4_09_g10730*) and TIFY 10b (*Macma4_03_g01960*). In *Arabidopsis*, SA synthesis is promoted by TFs SARD1 and CBP60g (*Calmodulin-binding protein 60g*), which regulate ICS1 (I*sochorismate Synthase*) expression and other immunity-related genes (Zhang et al., 2010; Wang et al., 2011; Sun et al., 2015). The detection of SARD1 (*Macma4_08_g16850*) in our study supports its conserved role as a key immune response regulator.

### 4.10 Plant hormones

Plant hormone signaling plays a pivotal role in defense responses. The SA pathway is activated upon recognition of pathogen effectors (*Avr*) by *R* genes, triggering downstream signaling that stimulate the induction of SA and the systemic acquired resistance (SAR) response against biotrophic pathogens. SA activation occurs through expression of EDS1, PAD4, and GDG1, as well as via MAPK signaling (Ding et al., 2022). Locally, SA induces HR and PR gene expression; systemically, it triggers SAR, a durable, broad-spectrum immune response in uninfected tissues that involves PR protein accumulation, lignification, ROS production, and antimicrobial compound accumulation, enhancing resistance against future pathogen attack (Klessig et al., 2018; Lukan and Coll, 2022). In this study, Salicylic acid-binding protein 2 (SABP2) (*Macma4_04_g03250*) and NDR1/HIN1-like protein 10 (*Macma4_09_g31980*) were upregulated in ‘Calcutta 4’ during early infection by *Foc* STR4. SABP2, an SA receptor, is essential for PR1 induction and SAR activation (Kumar and Klessig, 2003; Kumar et al., 2006; Vlot et al., 2008). NDR1 promotes SA accumulation (Shapiro and Zhang, 2001; Ding et al., 2022), with overexpression enhancing disease resistance in *Arabidopsis* (Lu et al., 2013).

Ethylene (ET) and jasmonic acid (JA) pathways, typically activated in responses to necrotrophic pathogens, mediate induced systemic resistance (ISR) (Li et al., 2019a; Ding et al., 2022). JA can induce the expression of PR proteins, peroxidases, alkaloids, volatiles, and structural defense mechanisms. Here, we observed both TFs MYC2 (*Macma4_07_g04870*) and JAMYB (*Macma4_09_g16730*) positively regulated at 2 DAI in ‘Calcutta 4’ after *Foc* STR4 infection. MYC2 activates JA-responsive genes (Du et al., 2017), while JAMYB enhances resistance to viral diseases in rice (Yang et al., 2020). Such upregulation of these TFs indicates a role of the JA pathway in the resistance response of this wild genotype to *Foc* STR4. Several ET-responsive TFs were also upregulated during the interaction of ‘Calcutta 4’ with *Foc* STR4 (**Supplementary Table S3**). ET receptors activate the EIN2 protein, which promotes ET signaling by inducing EIN and EIN3-like (EIL) TFs that bind ERF1 to stimulate ET-mediated defenses (Berrocal-Lobo and Molina, 2004; Dolgikh et al., 2019). In ‘Calcutta 4,’ ETHYLENE-INSENSITIVE 3-like 1a (EIL1) (*Macma4_08_g22960*) and Ethylene-responsive TF 1B (ERF1B) (*Macma4_11_g21380*) were positively regulated in response to *Foc* STR4.

Downstream defense responses following activation of JA/ET pathways typically involve the induction of PR genes, where ERFs function as key regulators of JA/ET-responsive defense genes (Huang et al., 2016). As ERFs are involved in immune responses in *Arabidopsis* to necrotrophic pathogens, with ERF1 overexpression enhancing resistance to *Botrytis cinerea*, *F. oxysporum*, and *Plectospherella cucumerina* (Huang et al., 2016), the role of ERFs in ‘Calcutta 4’ resistance to Foc STR4 merits further investigation.

### 4.11 Secondary metabolism

The SA, ET, and JA signaling pathways also regulate the synthesis and distribution of secondary metabolites during plant stress responses (Yang et al., 2019). Root-derived phytoalexin and flavonoid secondary metabolites exert antifungal activity by inhibiting or preventing spore germination, thus contributing to defense against phytopathogenic fungi (Agrios, 2005). Premnaspirodiene oxygenase, encoded by Cytochrome P450 (CYP71), confers resistance in rice to *Xanthomonas oryzae* pv. *oryzae* (Niño et al., 2016). This enzyme catalyzes regio- and stereospecific hydroxylation of sesquiterpenes to produce solavetivone, a potent antifungal phytoalexin (Takahashi et al., 2007). Stilbenes, another class of phytoalexins within the flavonoid family, are synthesized by Stilbene synthase (STS) which catalyses the final step of the biosynthesis (Jeandet et al., 2002). STS genes have been cloned in several plant species, including peanut (*Arachis hypogaea*), Scots pine (*Pinus sylvestris*), eastern white pine (*Pinus strobus*), Japanese red pine (*Pinus densiflora*), grapevine (*Vitis vinifera*), and sorghum (*Sorghum bicolor*) (Qayyum et al., 2022). STS enzymes are responsive to external signals triggered by abiotic stresses and biotic signaling from fungal cells (Jeandet et al., 2002, 2010). In this study, the genes Premnaspirodiene oxygenase (*Macma4_07_g21750*) and Stilbene synthase 1 (*Macma4_10_g24620*) were upregulated in ‘Calcutta 4’ at 1 and 2 DAI during *Foc* STR4 infection, contrasting with their absence in ‘Prata-anã’, as confirmed by RT-qPCR. These findings suggest an important role for phytoalexins in the defense response of ‘Calcutta 4’ against *Foc* STR4.

## 5 Conclusions

In the present study, we identified numerous DEGs positively regulated and involved in the immediate early stage defense response of ‘Calcutta 4’ to attack by *Foc* STR4. As summarized in the schematic model of the key findings of the innate immune responses observed in ‘Calcutta 4’ during interaction with *Foc* STR4 (**Figure 12**), these responses involve the activation of genes encoding PRRs, PR proteins, chitinases, NLR resistance proteins, TFs, and genes associated with ROS and SAR. Additionally, genes involved in phytoalexin biosynthesis, callose production, and the regulation of phytohormone signaling pathways such as ET and JA are also induced (**Supplementary Table S3**). Through histological analyses, we observed defense responses to *Foc* STR4 in ‘Calcutta 4’ involving callose deposition and accumulation of phenolic compounds which are likely responsible for the rapid resistance response in this wild genotype. In contrast, ‘Prata-anã’ exhibited a limited response to the pathogen, with histopathology and RT-qPCR data revealing an absence of phenolic compounds, callose and phytoalexins, suggesting that it lacks genetic machinery to activate resistance responses against *Foc* STR4. Functional analysis of DEGs supports the conclusion that the wild diploid ‘Calcutta 4’ mounts a rapid and robust defense response against *Foc* STR4. This response involves the activation of genes associated with stimulus perception and defense-related biological processes, as indicated by GO enrichment. KOG analysis further highlights the involvement of genes related to defense mechanisms and the biosynthesis of secondary metabolites. In parallel, KEGG pathway analysis reveals enrichment in plant–pathogen interactions, phytohormone signaling, and secondary metabolic pathways. MapMan analysis confirmed the involvement of genes related to hormone signaling, transcriptional control, and secondary metabolite biosynthesis. Of the candidate genes validated for expression by RT-qPCR, genes encoding proteins such as Acidic endochitinase, Peroxidase 4, Leucine-rich repeat receptor-like serine/threonine/tyrosine-protein kinase (PRR), putative Disease resistance protein RPS5 (NLR), Pathogenesis-related protein 1 (PR1), Thaumatin-like protein 1 (PR5), Stilbene synthase 1 and Premnaspirodiene oxygenase, which represent upstream to downstream defense responses, warrant functional validation via transgenic or CRISPR approaches.

Further research is warranted to functionally validate additional early defense-response genes and to elucidate the roles of later systemic or secondary defense mechanisms in this pathosystem. Candidate defense-related genes identified in ‘Calcutta 4’ represent valuable resources for the genetic improvement of susceptible banana cultivars, particularly those of the ‘Prata’ group, which are widely consumed in South America. These genes may be deployed through introgression or genome-editing strategies to enhance resistance to *Foc* STR4. Moreover, given the close phylogenetic relationship between *Foc* STR4 and *Foc* TR4, these defense genes also hold potential for conferring broad-spectrum resistance against both race 4 pathogens, which is of significant relevance for mitigating the global impact of Fusarium wilt on banana production.

## Data Availability

Data generated and analysed during the study is included in the article/**Supplementary Material**. Sequence data are deposited in the NCBI Sequence Read Archive (SRA) database as a BioProject, accession number PRJNA1198017.

## Supplementary material

Supplementary Figure 1 Kegg mapping of the MAPK (mitogen-activated protein kinase) signaling pathway based on differentially expressed genes (DEGs) observed in *Musa acuminata* subsp. *burmannica* accession ‘Calcutta 4’ after infection with *Fusarium oxysporum* f. sp. *cubense* STR4, at 1, 2, and 4 days after inoculation. Different shades of red indicate positively regulated DEGs (UP), while shades of blue indicate negatively regulated DEGs in the three treatments.

Supplementary Figure 2 Kegg mapping of the plant hormone signal transduction pathway based on differentially expressed genes (DEGs) observed in *Musa acuminata* subsp. *burmannica* accession ‘Calcutta 4’ after infection with *Fusarium oxysporum* f. sp. *cubense* STR4, at 1, 2, and 4 days after inoculation. Different shades of red indicate positively regulated DEGs (UP), while shades of blue indicate negatively regulated DEGs in the three treatments.

Supplementary Figure 3 Kegg mapping of the plant-pathogen interaction pathway highlighting the presence of differentially expressed genes (DEGs) observed in *Musa acuminata* subsp. *burmannica* accession ‘Calcutta 4’ after infection with *Fusarium oxysporum* f. sp. *cubense* STR4, at 1, 2, and 4 days after inoculation. Different shades of red indicate positively regulated DEGs (UP), while shades of blue indicate negatively regulated DEGs in the three treatments.
