# Supplementary figures and images for: Histological and transcriptome analysis uncover a robust early PTI and ETI-associated immune response in Musa acuminata subsp. burmannica accession ‘Calcutta 4’ to Fusarium oxysporum f. sp. cubense Subtropical Race 4
Source: Front Plant Sci. 2025 Sep 3;16:1621600. doi: 10.3389/fpls.2025.1621600 (PMC12441072; doi:10.3389/fpls.2025.1621600)

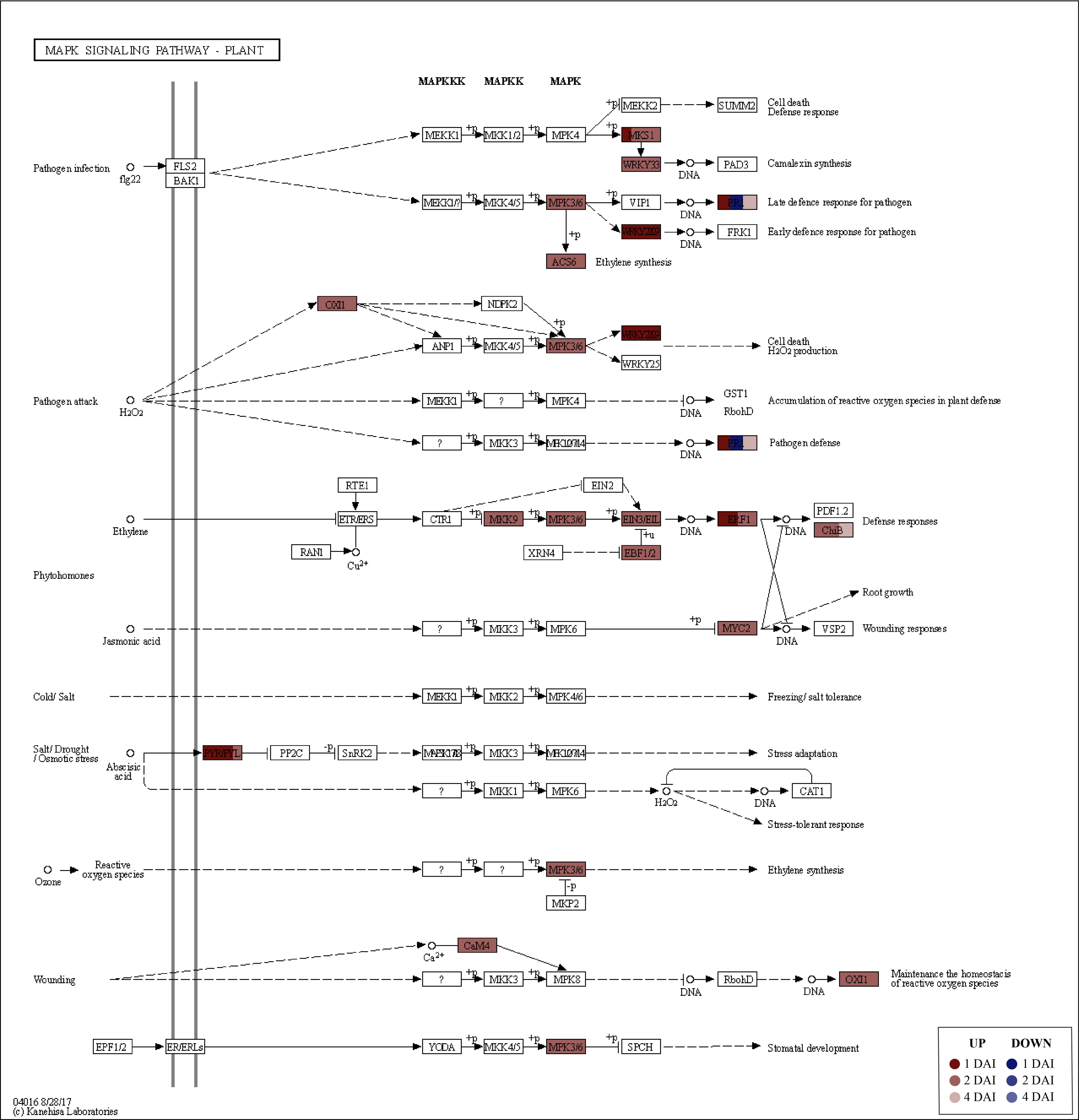

Supplement: Supplementary Figure 1 — Kegg mapping of the MAPK (mitogen-activated protein kinase) signaling pathway based on differentially expressed genes (DEGs) observed in Musa acuminata subsp. burmannica accession ‘Calcutta 4’ after infection with Fusarium oxysporum f. sp. cubense STR4, at 1, 2, and 4 days after inoculation. Different shades of red indicate positively regulated DEGs (UP), while shades of blue indicate negatively regulated DEGs in the three treatments. [file Image1.jpeg]

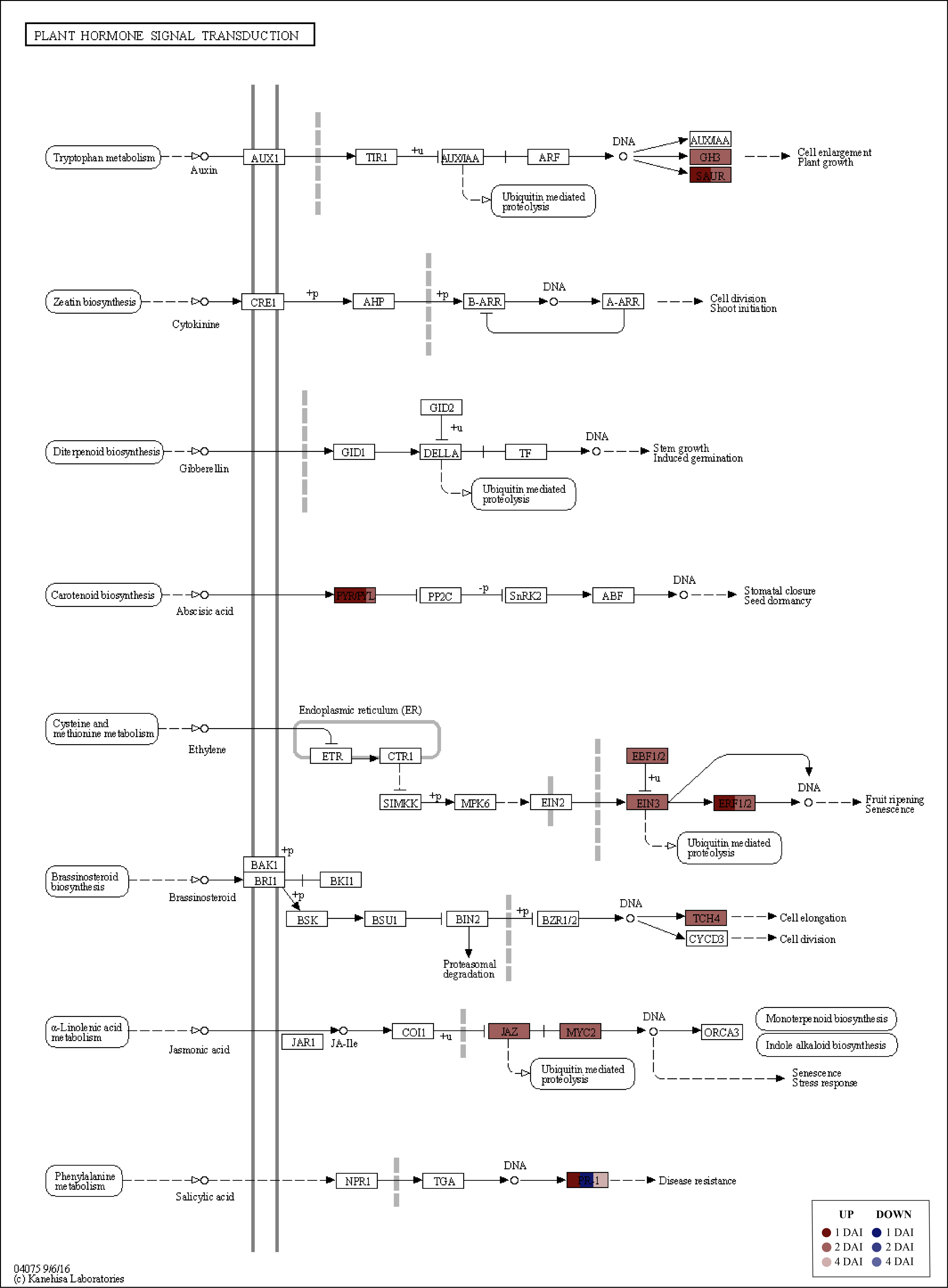

Supplement: Supplementary Figure 2 — Kegg mapping of the plant hormone signal transduction pathway based on differentially expressed genes (DEGs) observed in Musa acuminata subsp. burmannica accession ‘Calcutta 4’ after infection with Fusarium oxysporum f. sp. cubense STR4, at 1, 2, and 4 days after inoculation. Different shades of red indicate positively regulated DEGs (UP), while shades of blue indicate negatively regulated DEGs in the three treatments. [file Image2.jpeg]

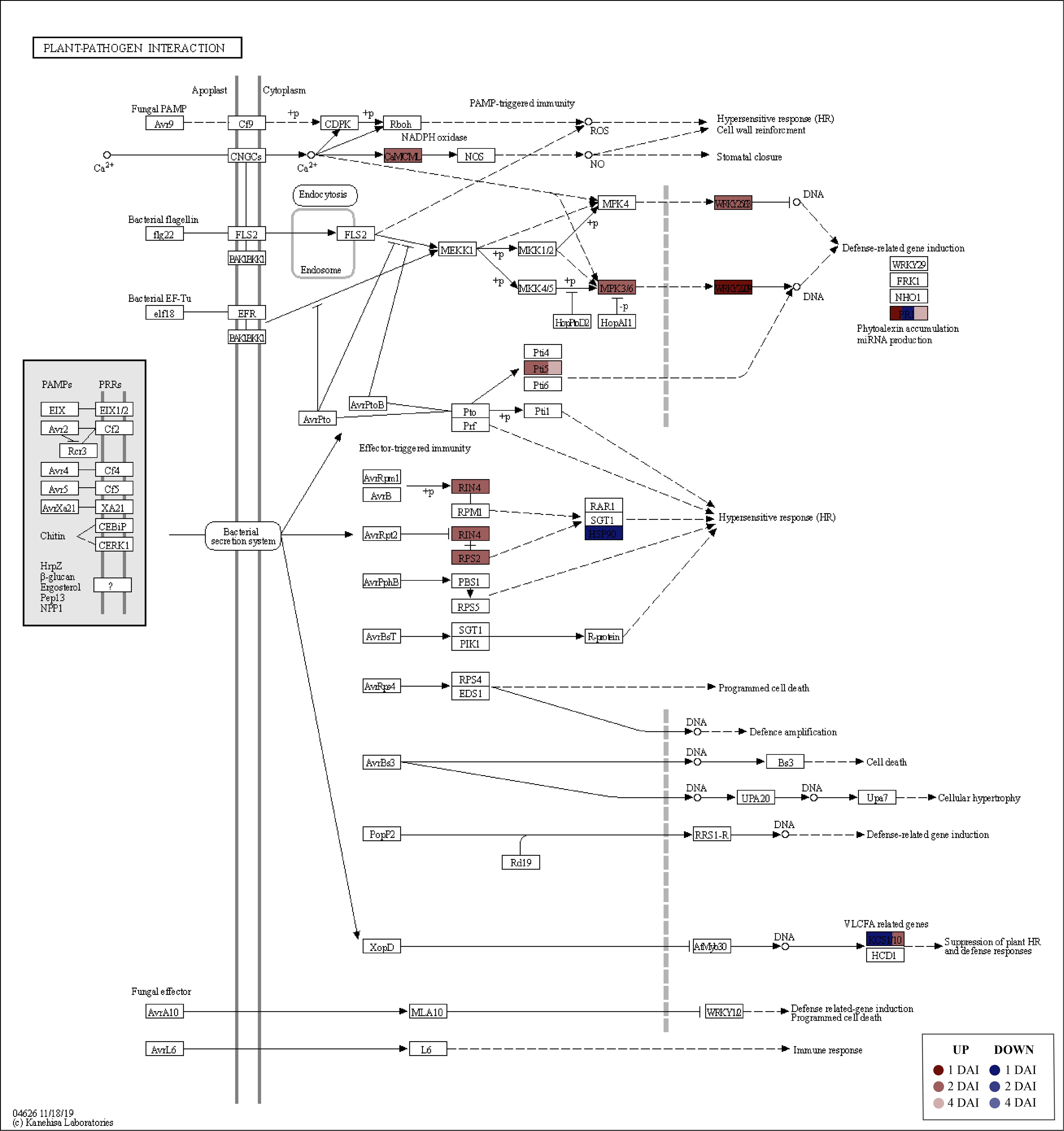

Supplement: Supplementary Figure 3 — Kegg mapping of the plant-pathogen interaction pathway highlighting the presence of differentially expressed genes (DEGs) observed in Musa acuminata subsp. burmannica accession ‘Calcutta 4’ after infection with Fusarium oxysporum f. sp. cubense STR4, at 1, 2, and 4 days after inoculation. Different shades of red indicate positively regulated DEGs (UP), while shades of blue indicate negatively regulated DEGs in the three treatments. [file Image3.jpeg]
